# Developing Visual Messages to Support Liquefied Petroleum Gas Use in Intervention Homes in the Household Air Pollution Intervention Network (HAPIN) Trial in Rural Guatemala

**DOI:** 10.1177/1090198121996280

**Published:** 2021-03-18

**Authors:** Mayarí Hengstermann, Anaité Díaz-Artiga, Roberto Otzóy-Sucúc, Ana Laura Maria Ruiz-Aguilar, Lisa M. Thompson

**Affiliations:** 1 Universidad del Valle de Guatemala, Guatemala City, Guatemala; 2 Laura Ruiz, Graphic Designer, Guatemala City, Guatemala; 3 Emory University, Atlanta, GA, USA

**Keywords:** audiovisual aids, behavioral change communication, health literacy, household air pollution

## Abstract

**Background:**

Household air pollution adversely affects human health and the environment, yet more than 40% of the world still depends on solid cooking fuels. The House Air Pollution Intervention Network (HAPIN) randomized controlled trial is assessing the health effects of a liquefied petroleum gas (LPG) stove and 18-month supply of free fuel in 3,200 households in rural Guatemala, India, Peru, and Rwanda.

**Aims:**

We conducted formative research in Guatemala to create visual messages that support the sustained, exclusive use of LPG in HAPIN intervention households.

**Method:**

We conducted ethnographic research, including direct observation (*n* = 36), in-depth (*n* = 18), and semistructured (*n* = 6) interviews, and 24 focus group discussions (*n* = 96) to understand participants’ experience with LPG. Sixty participants were selected from a pilot study of LPG stove and 2-months of free fuel to assess the acceptability and use of LPG. Emergent themes were used to create visual messages based on observations and interviews in 40 households; messages were tested and revised in focus group discussions with 20 households.

**Results:**

We identified 50 codes related to household air pollution and stoves; these were reduced into 24 themes relevant to LPG stoves, prioritizing 12 for calendars. Messages addressed fear and reluctance to use LPG; preference of wood stoves for cooking traditional foods; sustainability and accessibility of fuel; association between health outcomes and household air pollution; and the need for inspirational and aspirational messages.

**Discussion:**

We created a flip chart and calendar illustrating themes to promote exclusive LPG use in HAPIN intervention households.

Visual messages used with printed materials, such as flip charts, brochures, posters, and calendars are central to health education, enhancing delivery of behavioral interventions, increasing awareness and reminding people to engage, or disengage, in a specific behavior (Lipkus, 2007). Visual images may be more impactful than written messages, particularly among people with lower levels of literacy (Devakumar et al., 2018; Houts et al., 2006). However, an effective visual message needs to encompass the sociocultural context of people situated within their communities and be consistent with their perceived roles and practices (Chen, 1989; Jensen, 2012; Kreuter, 1999). Since notions are flexible and context dependent, we need to understand how individuals interpret visual messages about an idea or concept. Engaging people in the process of developing visual messages overcomes potential barriers that materials may not be understandable or applicable in a given setting or with a group of people.

Social science researchers use visual image methodology to elicit and interpret research findings (Bagnoli, 2009; Copeland & Agosto, 2012; Guillemin, 2004; Harper, 2002; Ross et al., 2009). Posters and calendars are two examples of visual methods that have been used to reinforce behavioral change communication, including the promotion of environmental messages. An Australian study cocreated a seasonal calendar with Indigenous communities to reinforce ecological messages promoting aquatic resource management (Woodward & Taggart 2019). Several household air pollution studies have used health education campaigns to reduce household air pollution exposures or to promote the use of cleaner cookstoves (Barnes et al., 2011; Devakumar et al., 2018; Jin et al., 2006; Oluwole et al., 2013; Tun et al., 2005; Zhou et al., 2006), but there are no publications describing the development of visual messaging tools. There is a gap in understanding how visual images represent community members’ understanding of household air pollution or beliefs about transitioning to new cookstove technologies (Evans et al., 2018; Hollada et al., 2017; Thurber et al., 2013).

In 2017, nearly 47% of the world’s population cooked with solid fuels, like wood. An estimated 1.6 million deaths are attributed to household air pollution annually (Health Effects Institute, 2019). Clean cookstove programs, including the provision of liquefied petroleum gas (LPG) stoves and fuel, aim to reduce indoor fine particulate matter to levels needed to meet the World Health Organization air quality guidelines of 10 µg/m^3^ (World Health Organization, 2014). However, long-term behavioral change leading to sustained use of clean stoves is challenging in rural poor communities where affordability and accessibility to clean fuels are primary barriers (Puzzolo et al., 2016). Messages about supporting the correct use of a new stove technology and fuel should be carefully aligned and effectively delivered to new LPG users. Visual messages that incorporate the promotion or avoidance of specific household behaviors can aid the adoption and sustained use of clean cookstoves (Goodwin et al., 2015; Thompson, 2018).

The Household Air Pollution Intervention Network (HAPIN) Trial is a multicountry randomized controlled trial of a LPG stove and free fuel distribution system delivered to half of the 3,200 enrolled households in India, Guatemala, Peru, and Rwanda. This trial aims to deliver evidence regarding health impacts in children during their first year of life, including low birth weight, stunting, and severe pneumonia, and blood pressure changes in older adult women residing in the homes (Clasen et al., 2020). This is an efficacy trial in which the investigators deliver and promote the uptake and consistent use of the intervention. Previous evaluations of improved cookstove have shown continued use of traditional stoves with the new stoves—a practice known as “stacking”—which is believed to vitiate the potential health benefits (Ruiz-Mercado et al., 2013). Behavioral intervention strategies that encourage households to adopt and exclusively use LPG stoves are essential for achieving the research aims of the HAPIN trial (Williams et al., 2020).

Before the launch of the HAPIN trial in April 2018, all four sites conducted formative research to develop behavioral messages that address household-level experiences with solid fuel and LPG stoves (Fandiño-Del-Rio et al., 2017; Williams et al., 2020). Three of the sites (all except Peru) conducted a pilot LPG stove and free fuel intervention over 2 months with 40 to 60 households to assess barriers to exclusive LPG use and to measure air pollution at baseline (with a solid fuel stove) and twice with the LPG stove. During our formative research in Guatemala, we invited 60 families to participate in this pilot study to create visual messages that would appeal to all household members in the intervention arm of the trial. The purpose of these messages was to inform participants about (1) the benefits of safe and exclusive use of LPG stoves and fuel and (2) the potential health and environmental effects of LPG use compared with firewood use. In Guatemala, we chose to design a flip chart to be used by the behavioral interventionist during the main trial and a wall calendar that will be given to each intervention home on the day the LPG stove was installed. The calendar was used to train household members on how to properly use the stove, as well as explain the benefits of cooking with LPG. Here we describe the process of creating visual messages for a flip chart and a calendar during our formative research in rural Guatemala.

## Method

### Procedures and Setting

The Guatemalan research site for the HAPIN trial is located in the Jalapa Department, approximately 2 hours southeast of Guatemala City. Rural communities in this area are primarily Xinca Indigenous people and speak Spanish. The altitude in these communities ranges from 871 to 2,677 meters above sea level, with a temperate climate that does not necessitate indoor heating (Clasen et al., 2020). The practice of relying on firewood cooking fuel throughout the year is common among rural Indigenous communities; poverty is the main driving factor, but traditional cooking practices that have been maintained and reinforced for centuries are also important (Thompson et al., 2018). The use of dung as a fuel is not practiced in Guatemala, unlike other low-income rural households, like in Peru (Fandiño-Del-Rio et al., 2017) and globally (Shupler et al., 2020). The primary sources of firewood are from peoples’ own lands and purchased fuel. This demand is exacerbated by woodstoves that require more wood fuel (Sharma & Dasappa, 2017), contributing to deforestation. Poorer households also use agricultural residues, as well as domestic trash, including plastic (Fedak et al., 2019). The different types of firewood depend on seasonal availability and accessibility, but households store wood and cook indoors or in outdoor shelters, thus they do not switch fuel use or cooking location during the rainy season. Alternatives to biomass fuels are still scarce and are determined primarily by wealth and accessibility (Puzzolo et al., 2016).

Local trained fieldworkers conducted brief verbally administered surveys of pregnant women who attended Ministry of Health prenatal clinics from 21 different communities in Jalapa to better understand the population that would be invited to participate in the HAPIN trial (Table 1). Among those, we randomly selected pregnant women with wood stoves using the same inclusion and exclusion procedures that would be used in the Main Trial (Clasen et al., 2020). Between June and August 2017, 40 households received an LPG stove and free fuel for 2 months to assess the acceptability and use of the intervention stove and fuel delivery system. In these households, we conducted participant interviews and observations. We took photographs of settings and people during their daily cooking activities to help us reflect on and evaluate how the intervention group would engage with the LPG stove and other cookstoves in the home.

**Table 1.** Characteristics of Women Receiving Prenatal Care From Ministry of Health Clinics (*n* = 292).

Demographics	
Age, years, *M* (*SD*)	25.8 (7.0)
Age in years, *n* (%)	
Women <18	32 (11)
Women 18–34	203 (69.5)
Women >34	36 (12.3)
Household members, *M* (*SD*)	6.1 (2.8)
Electricity in home, *n* (%)	206 (70)
Owns cell phone, *n* (%)	209 (72)
Active smoking, *n* (%)	4 (1.4)
No. of children <2 years in home, *n* (%)	67 (23)
Proportion of women to plan to migrate in following 6 months, *n* (%)	15 (5.1)
Primary cookstove use patterns	
Open wood fire, *n* (%)	169 (58)
Wood-fired chimney stove, *n* (%)	109 (37)
Liquid petroleum gas stove, *n* (%)	14 (4)
Pregnant women are main cook	209 (72)
Pregnancy characteristics	
Gestational age, *M* (*SD*) in weeks	16.6 (8.3)
Gestational age, *n* (%)	
<12 weeks	73 (25)
12–20 weeks	103 (35.2)
>20 weeks	176 (60.2)
Proportion of pregnant women eligible for enrollment in pilot study (18–34 years, <20 weeks gestation, use wood stove, primary cook, no plans to migrate, nonsmoking), %	44.5

We used social phenomenology (Desjarlais & Throop, 2011; Schutz, 1967) to explore the experience of exclusive use of LPG stoves, with the goal of visually representing these experiences. A medical anthropologist (MH) conducted qualitative methods using direct observation (*n* = 36 participants), in-depth (*n* = 18 participants) and semistructured (*n* = 6 participants) interviews to assess peoples’ cooking preferences and behaviors to understand households’ LPG use and underlying driving factors of the use of traditional wood-fueled cookstoves, including norms, values, preferences, and perceptions. Since current and future practices are related to previous knowledge and experiences, we explored perceived differences between wood and LPG stoves (see Supplemental Material for interview guides). These methods helped us identify 24 topics that became a thread, central to understanding sociocultural practices and perceptions of LPG use, which ensured local and cultural relevance as well as appropriateness. A Guatemalan graphic designer (ALMR-A) drafted images based on themes that emerged from this initial phase.

In the second phase of the research (September and October 2017), 20 additional households were recruited using the same procedures described above, except that these households also included an older adult woman residing in the home who also participated in the pilot study. With these households, we conducted 24 focus group discussions (FGDs) with 96 adults (all adults in the household were invited to attend) to review draft images representing the themes that emerged from the initial phase. Each FGD consisted of four participants. Based on previous experience, we limited men’s participation, because women in this setting tend to defer to men, who then speak more frequently and with more authority. However, in two groups, two husbands participated. Two groups validated the same four images to assure accurate representation for each image and to achieve homogeneity in interpretation of the images. All FGDs were conducted at one of the participants’ households and lasted 45 to 60 minutes. We asked participants to build interpretations of each image, allowing us to gather a range of perspectives. The following open-ended questions allowed for open discussion:

1. Please carefully observe the following four images. For each image:
  a. Is there something you do not like? What is it?
  b. What would you like to change/modify?
  c. Do you think that each image corresponds to the written phrase that describes the image? In your own words, describe what this image means to you:
2. What difficulties have you had with the gas stove? With the gas cylinders?

We aimed to achieve cultural consensus among participants even if some representations of an experience were “more-or-less” similar. Based on feedback from these groups, the graphic designer modified the visual messages.

### Data Analysis

All ethnographic notes and interviews were transcribed in Spanish and coded thematically by the primary author using HyperRESEARCH Software (Randolph, MA). Common themes, or code groups, that were mentioned repeatedly were grouped together using deductive and inductive approaches. For example, “fear” unified concepts of fear of getting burned, fear of explosions, fear of the children being near the stove and fear of not having LPG to cook. We developed networks from main codes to identify which concepts were closer or farther away to other themes. If the theme was too complex (e.g., “where does the gas come from?”), or too specific (e.g., “how do we handle the gas stove’s knobs?”), the theme was labeled as independent. We chose the themes that caused the greatest concern to new LPG users.

**Figure 1.**
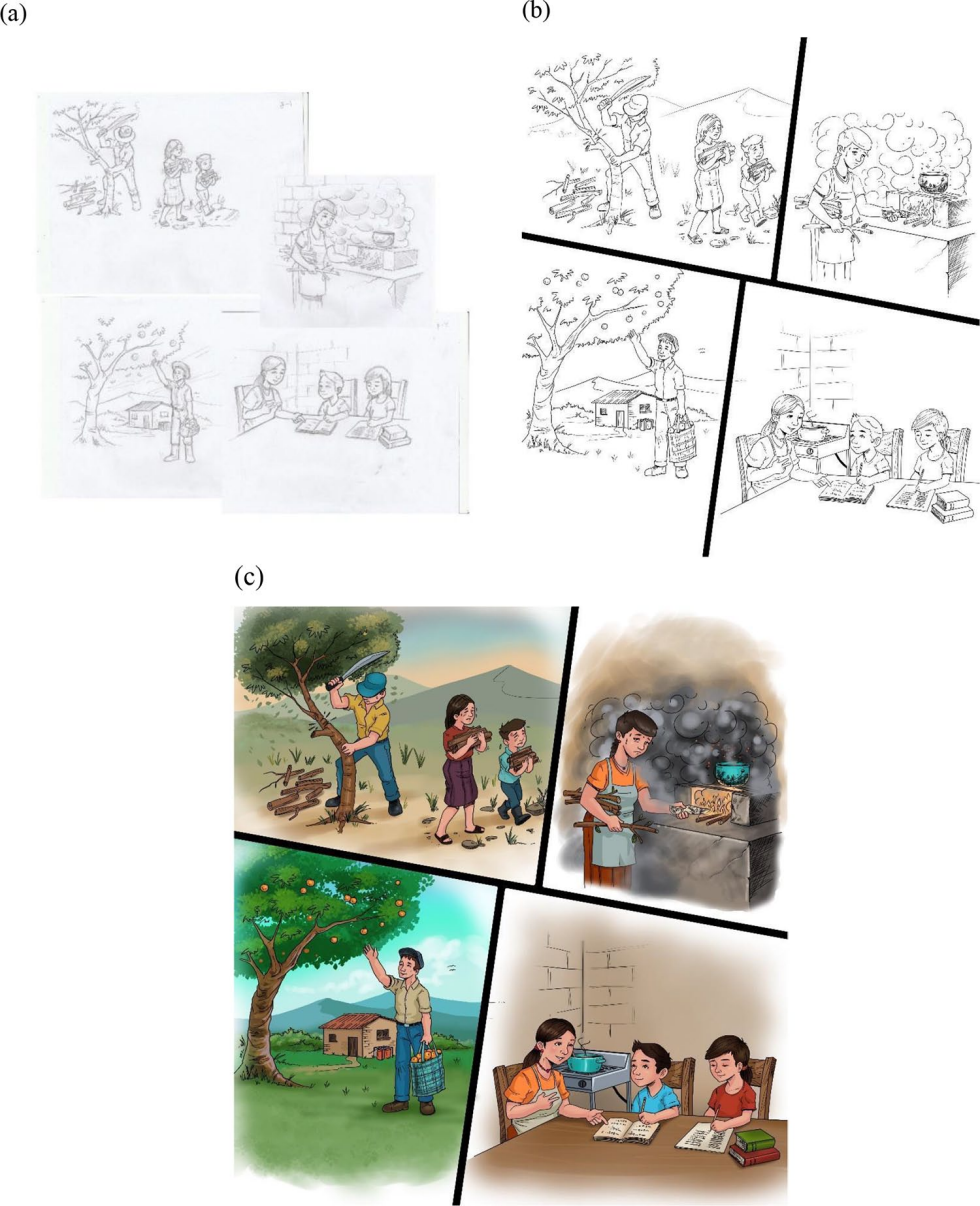
(a) Draft sketch shared with focus group. (b) Line drawing shared with investigators. (c) Final color image included in calendar.

For FGDs, one or more of our researchers (MH, AD-A, LMT) facilitated each discussion and a fieldworker (RO-S) served as a note-taker. We did not audio-record the sessions but transcribed the notes and relayed this information to the graphic designer (ALMR-A) who made modifications to the illustrations and written phrases in an iterative fashion based on participant suggestions (Figure 1). Clear and insightful comments given by participants during the qualitative data collection that helped elucidate constraints and meaningful aspects for the adoption and sustained use of LPG stoves were selected for each topic and illustration. Relevant quotes were those that better represented, supported, and clarified the messages to reinforce the visual messages in the flip chart and the calendar.

After coding the data, we created “descriptive images” that would represent the relevant themes or threads for the visual messages. With the help of photographs, descriptions, and quotes, the investigators (MH, AD-A, LMT) discussed the 50 codes we found. To avoid creating bias when sorting the data during the reduction process, we discussed whether the codes were related to LPG use, what the descriptive images represented, and whether there would be overlap or confusion about the images. During this process, the 50 codes were reduced to 24 themes that related specifically to sustained use of LPG use. The graphic designer (ALMR-A) elaborated 24 visual messages with help of photographs and participant descriptions. The first drafts were discussed again by the research team, to see whether the images represented the meaning and importance of the messages we wanted to transmit. With this feedback the graphic designer elaborated a more detailed second draft. These images were presented and discussed by the FGDs participants to identify issues of importance to them and to assure that the images accurately reflected the corresponding theme. To make certain that the images were correct and accurate, the final drawings were again revised by the research team before printing (see Supplemental Material).

### Ethics

The study protocol was reviewed and approved by institutional review boards or ethics committees at Emory University (00089799), Johns Hopkins University (00007464), Universidad del Valle de Guatemala (146-08-2016/11-2016), and the Guatemalan Ministry of Health National Ethics Committee (11-2016). All participants provided written informed consent.

## Results

Since practices are interrelated to previous knowledge and experiences, we linked ideas (past, present, and projections) that people have about cooking practices, as well as differences people perceive about wood and gas stoves. For that, we selected information from among families who were part of the pilot LPG stove study, choosing the most predominant aspects that caused the greatest concern to the new LPG users. We assessed these aspects as predictors or reasons to not use the gas stove exclusively during the HAPIN trial. Cooking practices that have been maintained and reinforced through years among families and communities cannot be expected to turn into commitments that contradict or devalue previous behavior. Therefore, it was vitally important to develop the material among people who are part of the same sociocultural setting. Since these families live in precarious economic conditions, their concerns associated with the staple foods they usually cook (beans and corn) is constant, that is, how it is prepared and how should it taste. We found that this created the idea of “traditional ways of cooking” to prepare these foods. The result of the method applied in our research showed how these subjective aspects have an effect on how people feel and their expectations of using a new stove. It also revealed people’s interest (or not) in cooking with gas; inferred how individuals become concerned with aspects related to health; and illustrated why (or not) this particular way of cooking is considered feasible.

The ethnographic research revealed more than 50 codes and subcodes based on peoples’ opinions, perceptions, and experiences that address their concerns, fears, doubts, and lack of understanding. Twenty-four themes were relevant to LPG use (Table 2 describes visual content for each of these themes) and were derived from these 50 codes. Each visual message was designed to portray a concept and realistically resemble the study area and participants. “Resemblance” aimed to help participants see themselves in the procedures, awareness, interests, responsibilities, and traditions. The illustrations depict differences between using LPG and biomass to ensure that each aspect of using an LPG stove fulfills not only expectations but also portrays a positive image of people’s social lives as well as their environment. Several important changes were the result of the focus group participants saying, “we don’t wear those clothes or shoes” or “we don’t cook with pots like that.” The complete set of 24 themes were developed into a flip chart that is used by the behavioral interventionist who visits intervention households to train families in intervention households on how to use the LPG stove use and fuel exclusively during the HAPIN trial. From the 24 themes, 12 were prioritized for the calendar based on frequency of themes mentioned and relevance to the purpose of promoting exclusive gas use. Each month portrays a goal-based concept. We narrowed down calendar themes based on participant relevance to important behaviors, such as fear or gas leaks, and notions that influence the use of LPG, such as cooking traditional foods like beans. This calendar was used by the behavioral interventionist to conduct the 2-hour initial training on how to use the stove and address the questions of the new user during the installation.

**Table 2.** Twenty-Four Themes That Promote Adoption and Sustained Use of Liquefied Petroleum Gas Stoves.

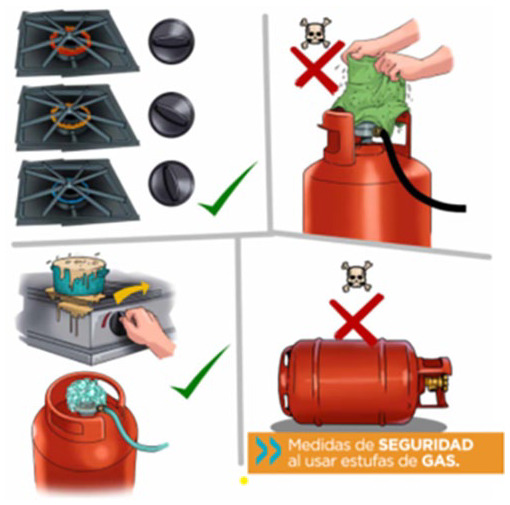	*Theme 1. If the necessary safety measures are followed, the cylinder and gas stove are very safe* **Message:** Although people fear gas explosions, gas use is safe. Explosions or accidents are caused by poor cooking practices and poorly maintained cylinders or hoses. To avoid this, we must follow safe procedures, which are easy to learn and remember: look for a blue flame; if water falls on the gas flame and it goes off, turn off the stove knob; do not put a wet rag on the cylinder, this does not prevent gas leaks; do the soap test if you suspect a leak; do not store the cylinder on its side and call the technical team if you do not know what to do or how to handle the cylinder. We must always remember to ventilate the area (open doors and windows) before turning on the stove, in case we suspect a leak. **Drawing:** Drawings (flame size, wet rag, pot boiling over, soap test, cylinder on side) with checks and X’s
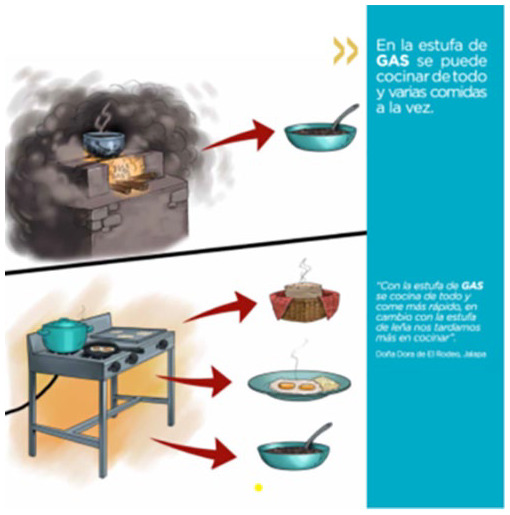	*Theme 2: Everything can be cooked on the gas stove and many foods can be cooked all at once* **Message:** There is a perception that certain foods have flavor when they are cooked with clay pots. But in reality, cooking processes can be changed such as using more or less oil or more or less water. We can soak beans before cooking them. This will reduce the cooking time and the amount of fuel. The use of condiments such as onions, garlic, pepper, and salt, gives food a pleasant taste. The palate can “readjust” its memory. **Drawing:** First drawing—smoky kitchen, one pot of beans cooking on high flame. Second drawing—Pots of different sizes with diversity of food (beans; tortillas; eggs) on the three burners.
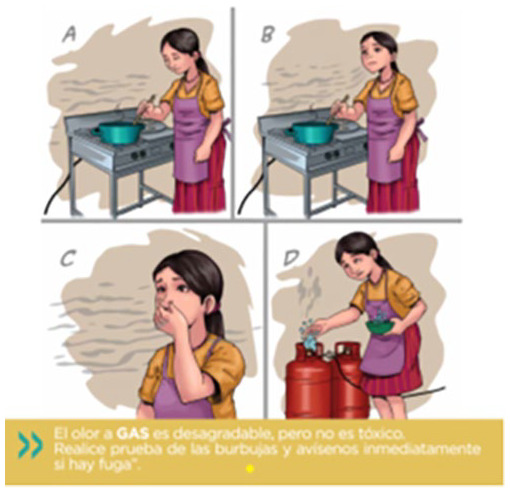	*Theme 3: The smell of gas can be unpleasant, but it is not toxic. Use the soap test and call us immediately if there is a leak* **Message:** Many people associate the smell of gas with something toxic. This odor is a substance called mercaptan. The companies that manufacture gas add it as a safety measure to quickly notice when there is a leak, or when one of the burners is left on. This substance is nontoxic, it only has an unpleasant odor. **Drawing:** Drawing of a woman smelling gas, recognizing a leak and performing the soap check to detect a leak.
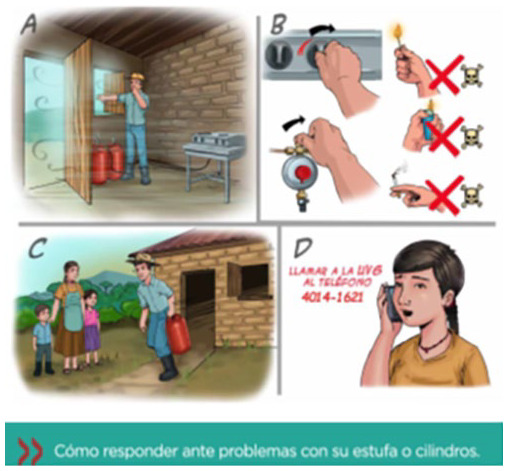	*Theme 4. How to respond to problems with the gas stove and cylinders* **Message:** HAPIN (Household Air Pollution Intervention Network) staff technicians will install the gas stove and two gas cylinders in the home. Household members will be taught by the technicians to switch the gas flow between the two cylinders, how to use the stove, including how to turn the gas flame on and off, how to use the spark lighter that will be provided, and how to cook with gas. We will also give instructions on how to detect and respond to leaks from the cylinder or stove. In case of a malfunction, the family will communicate with the technical support staff who will come to review the stove and cylinders. **Drawing:** Show a man responding to a situation of risk (gas leak) 1. In case of suspected leak, switch off the stove and close the valve 2. When the cylinder is inside the kitchen or a room, open all windows and doors to ventilate the room 3. DO NOT light matches, cigarettes or any other item to make fire 4. In case the cylinder is inside the kitchen, disconnect the cylinder and take it outside Call us on the phone
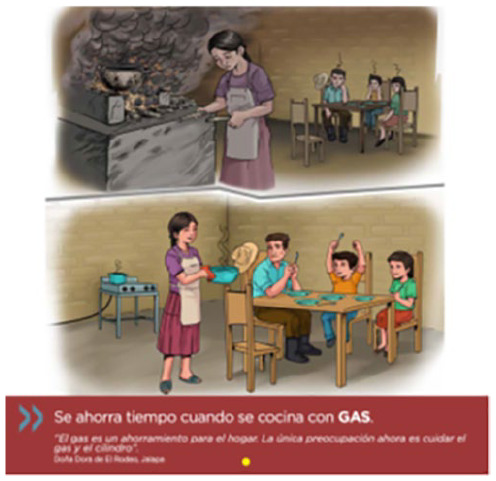	*Theme 5. I save time when I cook with gas instead of wood* **Message:** The delivery of the stove will be accompanied by two 25-pound gas cylinders. When the first cylinder runs out, the family should switch the valve to start using the second cylinder and call the study team using the toll-free number provided to them. The study team will deliver a replacement of the empty cylinder within 2 days. **Drawing:** First family: A woman cooking with firewood (with a hungry family waiting to eat). Second family: woman serving hot food (with the family ready to eat)
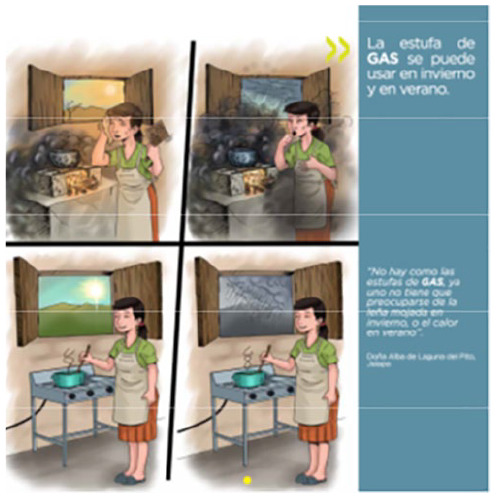	*Theme 6. I can use the gas stove during the summer and the winter* **Message:** Avoiding the use of firewood will prevent the kitchen area from getting too hot during the summer months. During the rainy winter months, using the gas stove avoids using wet firewood that produces a lot of smoke. **Drawing:** First set of pictures: A very hot woman sweating while cooking in the summer months. The same woman with discomfort caused by the smoke generated by wet wood used during the rainy season. Second set of pictures: Show the same woman cooking without problems and discomfort during the dry and rainy season. The room is cool and there is no smoke.
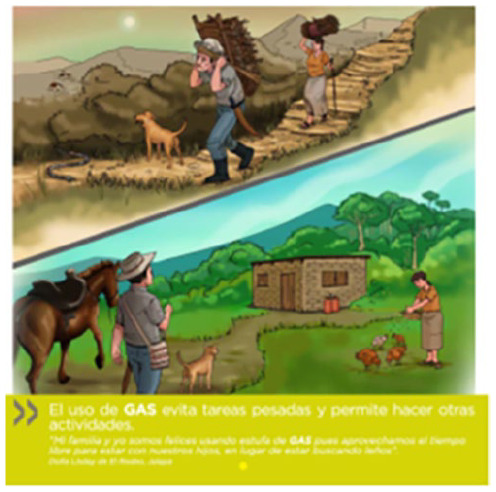	*Theme 7: The use of gas helps me avoid cumbersome tasks and allows me time for other activities* **Message:** Collecting firewood involves activities such as going out frequently to search, cut, collect and transport firewood, travelling long and dangerous distances carrying heavy loads of wood. During the summer, this is made difficult due to excessive heat and during the winter, by heavy rains. Snake bites are common and dangerous. With gas, we no longer occur have to gather wood. **Drawing:** First drawing: A man and a woman carrying wood on their backs and heads as they walk steep trails with a snake traversing the path. Second drawing: A man walking back from his plot to his house without a wood load. House has two gas cylinders outside and a woman feeding chickens.
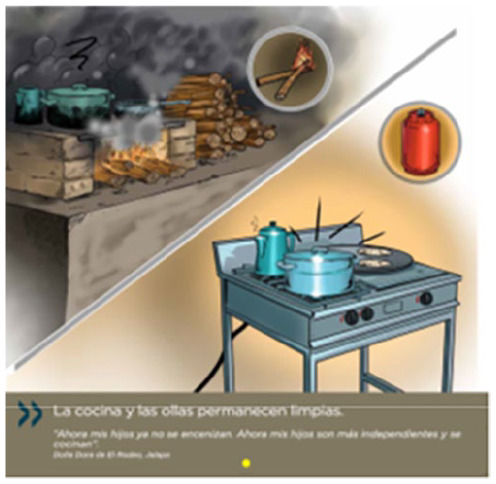	*Theme 8: The kitchen and pots and pans stay clean* **Message:** When using wood for cooking, the pots blacken and are very difficult to clean. This means that it is hard to keep the kitchen looking clean and the pots deteriorate over time. Using gas stoves avoids this, even if clay pots are used. The pots stay cleaner and last longer. **Drawing:** First picture: Stained pots damaged by ashes. Second picture: Clean, well-maintained pots on a gas stove.
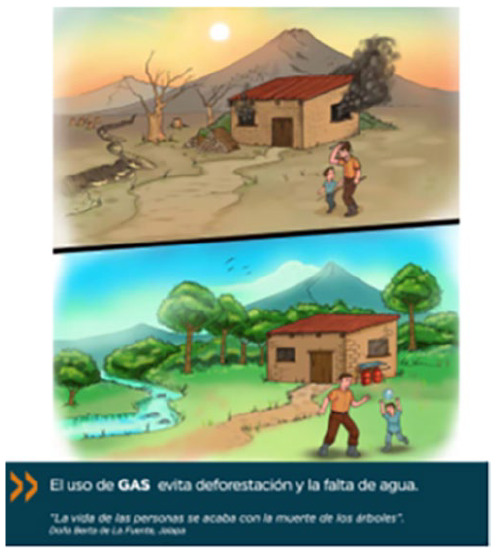	*Theme 9: The use of gas prevents deforestation and lack of water* **Message:** By consuming firewood every day we are contributing to deforestation. With the passage of time, it will be more difficult to find firewood, causing more droughts and lack of water, aspects that have a direct impact on our well-being, health and economy (corn and coffee crops). **Drawing:** First picture: A home with smoke coming from the window (indicating wood use) with a dried-up river, hot sun, and deforested mountains. Second picture: Home with gas cylinders, a blue river, green trees, father and son playing on grass.
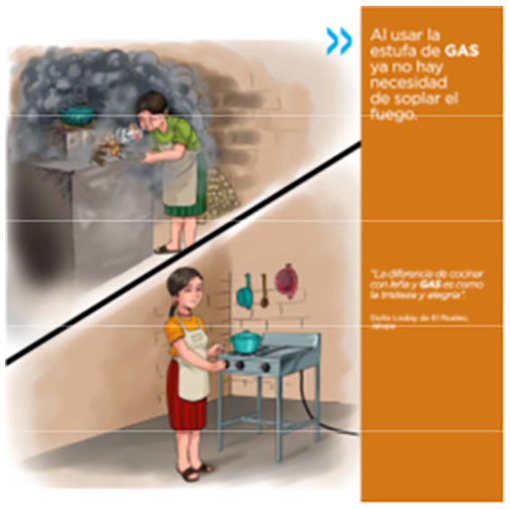	*Theme 10: There is no need to blow on the fire when using the gas stove* **Message:** The use of gas prevents women or children from blowing on the fire to stoke embers. This is not only an annoyance and can be arduous (especially when the wood is wet or freshly cut), but also prevents breathing in smoke and prevents ashes from falling into the food, clothing, and the kitchen. **Drawing:** First picture: A woman struggling to light the fire, which is constantly extinguished, causing more smoke and discomfort, as well as delaying the cooking process. Second picture: A woman easily igniting the gas stove.
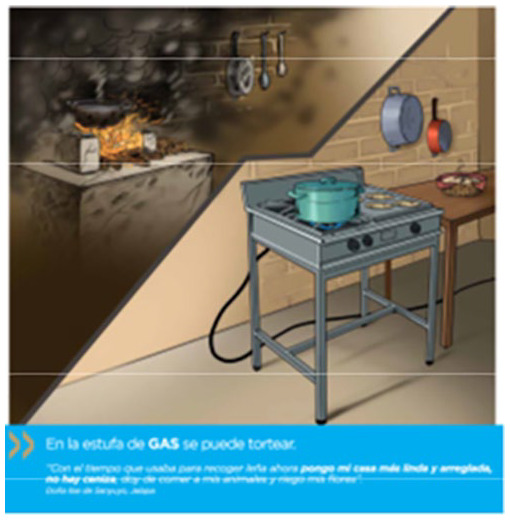	*Theme 11: You can use the gas stove to make tortillas* **Message:** The stove has three burners, one of which is large and contains a flat metal comal, or *plancha*, to make tortillas. However, if you prefer to use clay comales, you can dismantle the metal comal and place a clay comal about 16 inches in circumference. **Drawing:** First picture: clay comal on a cracked raised open fire (polletón) with photos of chicken dish and plates, accentuating the crack of this stove. Second picture: built-in comal on the gas stove.
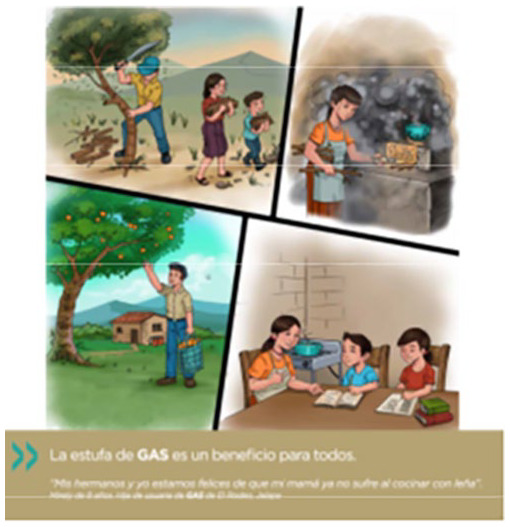	*Theme 12: The gas stove benefits everyone* **Message:** The prolonged use of the gas stove has various impacts on personal, family and community life. Some of these can be direct and personal, such as more time for other activities and less discomfort when cooking; others indirect, such as a cleaner home; but can affect the community, since we conserve natural resources of the area and keep the air cleaner when we don’t burn wood. **Drawing:** First set of pictures: Each member of the family involved in an activity dedicated to the use of firewood (extraction, cutting, loading, cooking). Second set of pictures: A mother helping with homework for the children and a man picking fruit from a tree.
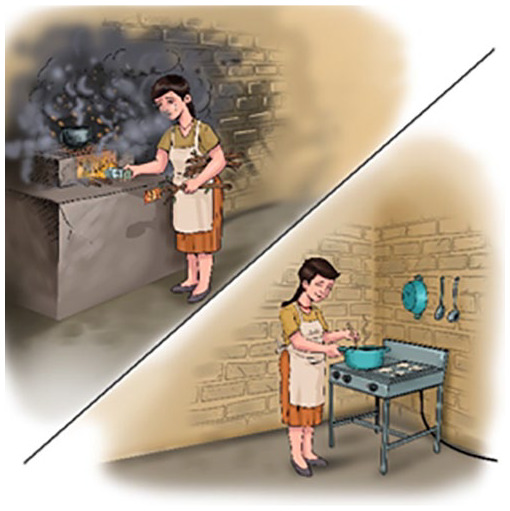	*Theme 13. Burning trash and using green wood in the cooking fire is toxic for health. Using a gas stove helps us avoid trash fires and using green wood in the kitchen.* **Message:** When using gas, we avoid burning garbage like plastic (e.g., bottles of soda, chip bags, etc.) in the cooking fire. Green wood, or uncured wood, is very smoky. This smoke is highly toxic to our health and also contributes to environmental, or outdoor, pollution. **Drawing:** First picture: A woman putting garbage on the fire, and “green” firewood (freshly cut or wet), which causes a lot of smoke and discomfort. Second picture: A woman cooking with gas without discomfort
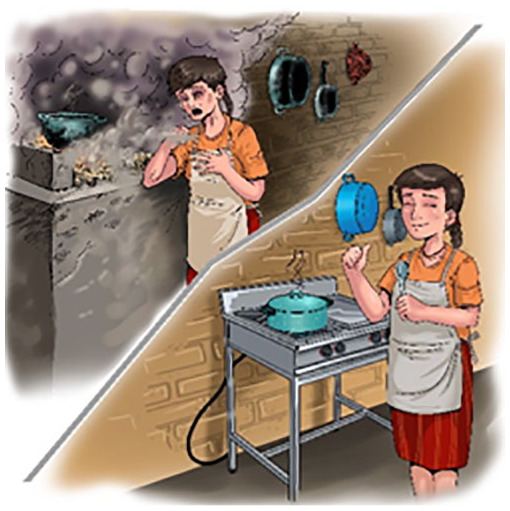	*Theme 14: Wood smoke is harmful even though it comes from trees* **Message:** Although wood is of plant origin, when burned it produces toxic substances similar to those produced by cigarettes, and this smoke affects our respiratory system. These inhaled substances cause many respiratory problems. The most vulnerable groups are young children, women and the elderly. **Drawing:** First picture: A woman inhaling wood smoke and coughing. Second picture: Same woman, without symptoms, using a gas stove.
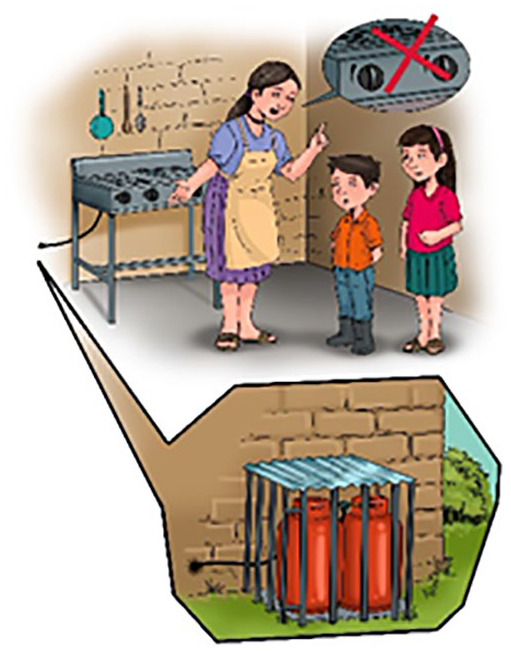	*Theme 15: Precautionary measures keep our children safe* **Message:** Just as we protect young children from approaching the wood fire, children should also be taught that a gas stove and cylinder are not toys and should not be touched. To avoid incidents of this type it is recommended (1) to educate the children to stay away from the stove, (2) to place the cylinder outside if possible (not exposed to rain and sun), (3) to place the cylinder inside a padlocked area if possible. The latter also prevents cylinder theft. **Drawing:** Mom educating the children not to manipulate the stove knobs. Gas cylinders located on the other side of the wall in a padlocked cage with protective aluminum roof.
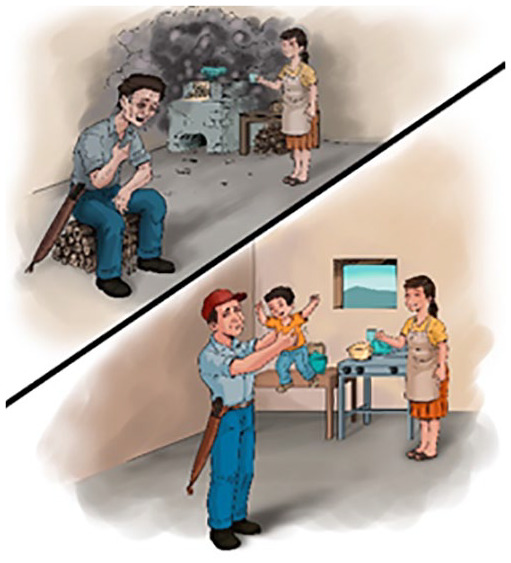	*Theme 16. The smoke produced by firewood affects everyone* **Message:** Although there is a perception that smoke sickens some, while only causing discomfort to others, all inhabitants of households that use firewood suffer consequences caused by smoke. Smoke is harmful even if we don’t see it, since the particles produced during combustion spread throughout the house. It does not matter if someone is considered strong or resistant to smoke. Everyone is vulnerable to the negative effects of smoke. **Drawing:** First picture: A family that uses wood for cooking, where the father is sick Second picture: the same man, healthy and playing with children, with a woman cooking with gas
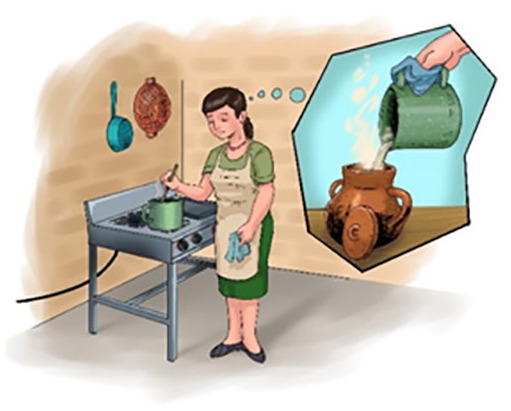	*Theme 17. Food can remain hot if we use certain types of pots* **Message:** Clay pots are efficient, allowing rapid heat transmission and long heat conservation. That is why when the fire is extinguished, the food remains hot longer. In this way we could cook with metal pots and then place the contents in clay pots to keep food hot longer, if we want to avoid re-lighting the gas stove. **Drawing:** A woman making porridge in a metal pot on the gas stove and pouring it into a clay pot
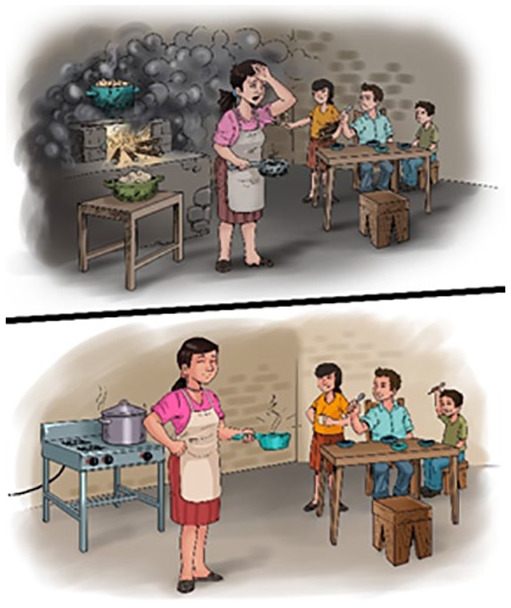	*Theme 18. Cooking with firewood is NOT part of our culture* **Message:** There is a perception that things are done because it is part of culture—practices or ideas that are transmitted from generation to generation. But often these practices or ideas are habits, conditioned by our socio-economic environment. Cooking with firewood is one example. The use of firewood is predicated by other conditions, such as easy access to firewood and lack of money or opportunities to purchase gas stoves and cylinders. Is the wood stove part of our cultural heritage? If it allows us to strengthen social bonds, promote important values such as unity and solidarity, becomes our cultural identity, then yes. But more often than not, the use of woodstoves is due to lack of economic opportunities. **Drawing:** First picture: A woman cooking beans and making tortillas or preparing tamales is a wood stove, tired and sweating. Second picture: A woman doing the same on a gas stove.
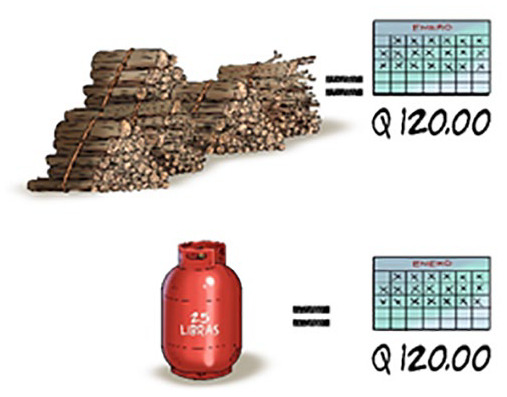	*Theme 19. The use of gas saves money* **Message:** Some think that the expenditure on fuelwood is lower than the expenditure on gas. One of the factors that perpetuates this idea is that fuelwood is cheaper because it is paid for small rations. But when converting the total fuelwood payment over a month, wood costs are higher than gas payment. **Drawing:** Shows a lot of wood accumulated in the house with a monetary equivalent versus a single cylinder and its value.
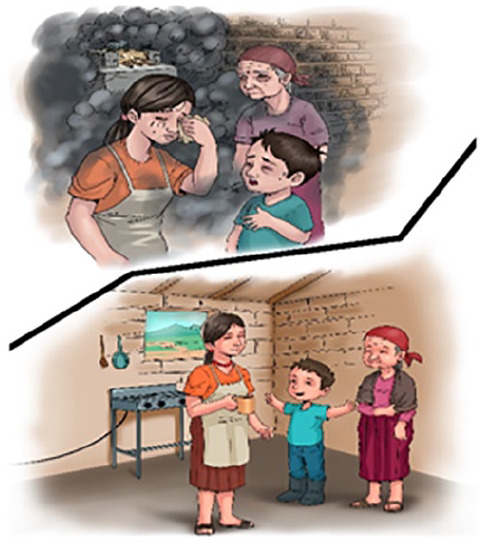	*Theme 20. The continuous use of the gas stove prevents discomfort/illness/accidents* **Message:** Frequent and prolonged use of firewood and exposure to woodsmoke can cause illnesses or discomfort, and even death. Many times, we associate diseases with other causes, or we do not know their origin. Pneumonia in children and adults, chronic cough, chronic lung disease, lung cancer, cataracts, cardiovascular disease, premature birth and low birth weight may be some of the diseases caused by exposure to smoke. The risk of burns also increases with woodstoves. **Drawing:** First picture: A sick family, each suffering from a different disease (boy with cough, mother with burning eyes, grandmother with cataracts). Second picture: Same family, healthy, with a gas stove.
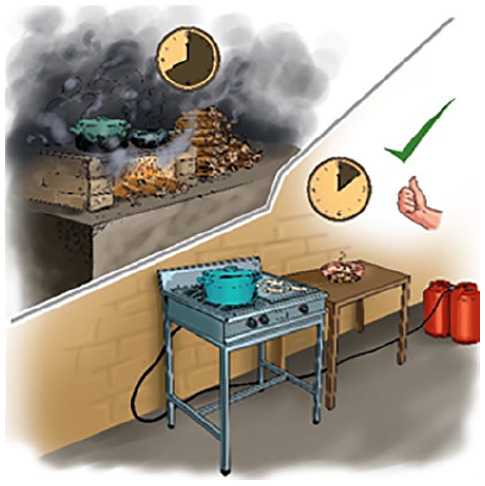	*Theme 21. Keeping foods hot depends on the material of the pots in which they are stored. You can quickly reheat foods after cooking them on the gas stove. This is different than wood stoves, where you have to continually feed the fire throughout the day to keep the fire and the food hot.* **Message:** The plancha stoves have a large surface to make tortillas, and you can leave several pots on the plancha after they have been cooked, to keep food warm. However, this requires stoking the fire throughout the day to keep the surface warm. In addition, tortillas are made before or after a mealtime is over. The gas stove has enough burners to cover all needs. We can learn to make efficient use of the gas stove, reheating food when we want to eat. The gas stove is “on/off” while the plancha stove needs embers all day to fire up during cooking times. **Drawing:** First picture: Several pots on the plancha with a clock showing 8 hours (time the fire has remained on) next to a tower of logs. Second picture: Several pots and a comal on the gas stove, with a cylinder that indicates a minimum use of gas, and the same clock showing the lapse of two hours.
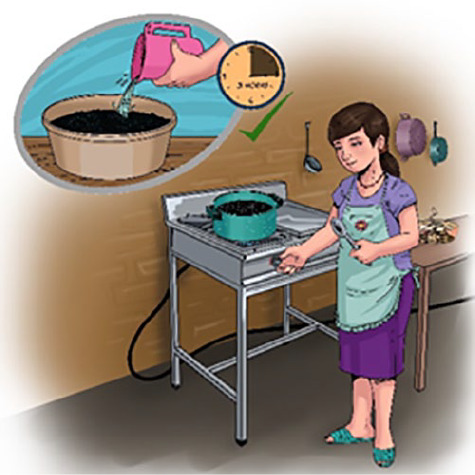	*Theme 22. I will never be without the possibility of cooking* **Message:** The stove will be accompanied by two 25-pound gas cylinders. When the first cylinder runs out, the family should call the study team to the toll-free number so that we can replace the empty cylinder. Many people use the gas stove only to cook foods that are quick to cook (such as eggs, precooked rice, coffee) or to reheat food. However, the functionality of the gas stove is the same as that of a plancha or open fire. You must learn to regulate the flame of the stove and look for the most suitable pots so that the combustion of the gas is more efficient. **Drawing:** Woman cooking beans on the gas stove. An inserted drawing where the beans are soaking, then being placed on the stove at low flame.
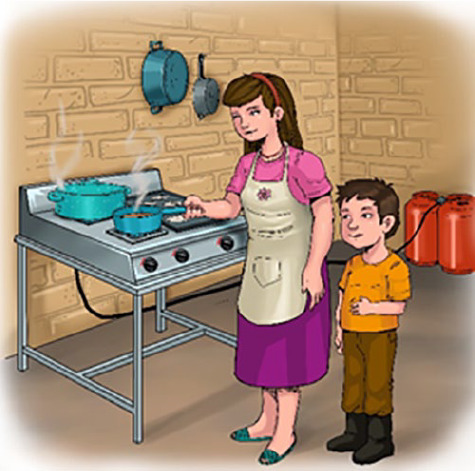	*Theme 23. On the gas stove you can cook everything, we only need to learn how to adjust the flame, which is easier to control than putting logs of various sizes and thickness* **Message:** The stove allows us to make use of different types and sizes of pots, even clay pots. It is important to regulate the flame of the gas stove. The flame is not only “very weak” or “very strong,” there are, between these two levels, other gradient levels of flame. **Drawing:** A woman cooking with pots of different sizes on the three burners.
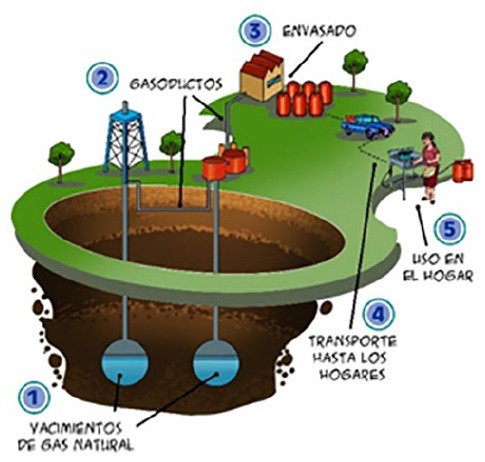	*Theme 24. Where does gas come from?* **Message:** Gas is a natural fuel (i.e., it has not been created by man), composed of a mixture of hydrocarbons (substances formed by carbon and hydrogen), of fossil origin (petrified remains of something previously living, like plants, which died billions of years ago) that are found underground. Gas is a nontoxic, colorless, odorless, and lighter product than air. Once extracted from the subsoil gas it is transported through gas pipelines. **Drawing:** A drawing illustrating the process of gas extraction and bottling. This gas cylinder is then shown placed near the stove of the study woman.

*Note*. First 12 themes formed the calendar messages.

### Fear and Reluctance

Two main themes that appeared consistently and that are crucially important to overcome are fear and reluctance (Table 3 shows representative quotes from the FGDs). A common theme was fear of explosions—a draft illustration portrayed this theme. Yet, the focus group perceived the image negatively, and were alarmed by the danger, so the image was redrawn to illustrate what to do to avoid gas leaks (Table 2, Theme 1) and what to do if there is a gas leak (Theme 3). Positively framed themes support self-confidence and awareness about safe LPG use.

**Table 3.** Representative Quotes From Focus Group Discussions for Calendar Themes.

Theme	Quote 1	Quote 2	Quote 3
Fear and reluctance	*. . . you always hear stories about explosions, cylinders exploding and people getting burned. A couple of months ago there was news about it somewhere, I can’t remember where it was, but a woman was severely injured because when she tried to turn on the stove, the cylinder exploded and she ended up at the hospital . . . I don’t know if she died or not but the photo showed how the kitchen was turned into debris.* (Female participant in *Anshiguan*)	*The reason why I always think it’s not safe to have a gas stove at home is that children are naughty, even when you tell them not to touch something they do it behind your back. My sister-in-law has a stove, a small one of two burners but she doesn’t use it because of that, she’s also afraid that her daughter will open the knobs, especially little children that don’t recognize dangers.* (Female participant in *Achiotes Jumay*)	*My youngest son is only 15 months but with this (wood) stove I’m not afraid that he’s going to get burned because it gets so hot that he doesn’t come close . . . but with a gas stove I would be afraid because he wouldn’t notice how hot it is, so a pot or a pan can just fall on him, that’s why it’s not safer.* (Female participant in *Chagüite*)
Traditional cooking practices	*I cook beans every two or three days because the pot is big but I make tortillas every day. When I prepare beans, I put the pot early in the morning so at midday it’s ready. I can leave the pot on the (wood) stove with just a few pieces of wood while I take the corn to be ground. Beans need to be prepared in clay pots for a few hours. When I come back home the stove will be hot enough to start making tortillas . . . We eat fresh tortillas every day; when you reheat tortillas these are not so tasty anymore, you need to toast them. If I have time, I make tortillas twice a day. I don’t like to buy tortillas at the stores women use gas to make them so they don’t taste good. My husband doesn’t like them either*. (Female participant in *El Arenal*)	*I worked in a kitchen for a few years where we only used gas stoves, that’s why I know how to cook with one of these, but I prefer cooking with wood because beans, tortillas and atole [porridge drink] taste better when prepared in clay pots on wood (stoves)*. (Female participant in *Miramundo*)	*We sell tamales or chuchitos [type of tamal] every Saturday or when someone asks us to prepare for a festivity or funeral. I use an open fire because the pot is quite big, a heavy clay pot. You have to cook the tamales with such pots, like our elders used to. Tamales taste much better when you prepare them in the traditional way, that’s why people like to buy tamales from us . . . Tamales need to be simmered for many hours and let them be covered with plantain leaves and you cannot do that with another (type of) stove. Depending on how many tamales you are cooking you just adjust the wood. All these things cannot be done with a gas stove. Gas stoves are good if you want to cook something quickly.* (Female participant in *Sansayo*)
Wood versus LPG	*Sometimes I help my little children with their homework but I can’t always sit and see what they have to do because when you are cooking you need to be attentive all the time, paying attention that (the food) doesn’t get burned, stirring (the food), especially if it’s atole you are preparing. Maybe if one could cook swiftly, then one could have time to do more things, more time for the children or for themselves, do other things or just rest.* (Female participant in *El Lazareto*)	*I tell my husband “take the children with you so they can help you” but he doesn’t like it because he says that there (where he cuts the firewood) are snakes, “better not” he says, “it’s better if they stay here at home helping (you).” Many people from this area have been “stung” (bitten) by snakes. They are long and black and it seems that they are poisonous. I didn’t know, but he told me so, then better not, better they (the children) stay at home playing*. (Female participant at *Pino Zapotón*)	
Sustainability and accessibility	*We pay 200 quetzales [USD25] for the firewood we need each month, my husband picks “two loads” each week with a horse. But if we don’t have money to buy all the wood that we need, we always are able to gather small pieces of wood on the land where we cultivate coffee, beans and maize. Every three years you need to cut the coffee trees because they don’t produce anymore. This wood is really good for cooking. So, we always have a way to find wood. But you cannot do that with gas. If you don’t have money you can’t buy a cylinder, so you can’t cook and you can’t say “I won’t eat until I have the money to buy (a cylinder).”* (Female participant in *Las Flores*)	*I remember that when I was little we were able to see trees everywhere. But most of this is gone. We have a protected area and you need a special authorization to cut branches there, but nowadays there are too many families living here. Now we have problems with not having water, not having wood. We are cutting more than planting trees. We once had a gas stove but we never used it. There is no money, no jobs, so what are the options?* (Male participant in *Azucenas*)	
Health outcomes	*We are used to the smoke, so I can’t tell you if it’s good or bad. I’m over sixty and I have cooked with firewood my whole life, as my mom did. Sometimes your eyes burn but this happens when the wood is wet or green, that’s why you have to use branches from trees that don’t produce too much smoke. We have a “plancha” [stove] with a chimney and all the smoke goes out; you don’t even notice the smoke.* (Female participant in Laguna Verde Abajo)	*. . . well yes, sometimes I have some (health) problems, like coughing or a bad cold, but that’s because I’m old. All my ailments are because I’m old. Look, children don’t get sick as often as old people do because they are still young or if they do they recover more rapidly . . . I have never heard before that someone died because she had cooked with firewood. If that were true, then we all would be sick, right?* (Female participant in *El Arenal*)	
LPG is “not natural”	*Gas is toxic, you can tell by how it smells, even food tastes bad when you cook with gas that’s why I don’t like it, I’m afraid we will get sick if the food gets poisoned with gas.* (Female participant in *Llano de la Puerta*)	*The smoke is like rain for everyone, but gas is not only dangerous but also toxic. I wouldn’t like my spouse using gas for cooking. I also don’t like microwaves. All those “new things” just pose a danger to our health, you don’t know if they can give you cancer, it’s better to use. what’s natural.* (Male participant in *Azucenas*)	*Cooking with firewood is not easy and you can get aches and pains but this is nothing compared to gas, because (while) the wood comes from trees, the gas is produced in big industries and put into cylinders. You can even smell it. That can’t be healthy, that can’t be good for you.* (Female participant in *Chagüite*)

*Note.* LPG = liquid petroleum gas.

Another common theme identified was young children playing with the stove knobs or coming too close to the stove, which could result in gas leaks or burns. People were therefore reluctant to use this new technology. To communicate these risks (Lipkus & Hollands, 1999), a visual message depicts how to avoid accidents when using the LPG stove (Table 2, Theme 15).

### Traditional Cooking Practices

Traditional cooking practices, maintained and reinforced for generations, cannot be expected to change if new behaviors are viewed as contradictory to previous behaviors. The visual material is based on a contemplation phase (reflecting about the behavior, i.e., the process of awareness) and the preparation and action phase (Prochaska & Norcross, 2001). Cooking staple foods using the “traditional ways of cooking” was an important theme (Table 3). We found that when culinary practices revolve around a few staple foods, like corn and beans, people appeared reluctant to change their ways of cooking. We observed that people described the type of food they cooked, directly associated with ideas or perceptions about *how* this food should be traditionally prepared (Table 2, Themes 2, 11, 17, 18, 21, and 23).

### Wood Versus LPG

We identified positive messages that support new behaviors around LPG use, and used an integrated strategy to contrast cooking with the traditional wood-stove or LPG stoves (Table 3). We aimed to demonstrate a relationship between new cooking behaviors that affect all members of the family depending on what role members play in the household (Table 2, Themes 5, 6, 7, 10, 12, 13, and 16).

### Sustainability and Accessibility

“Sustainability” and “accessibility” of fuel were recurring themes (Table 3). The HAPIN trial will provide free fuel, so we addressed the concern of not being able to cook if they ran out of LPG (Table 2, Theme 22). Participants who gathered wood expressed concern about the effects of deforestation in rural areas where there are no measures to protect forests or plant new trees (Table 2, Theme 9). For participants who purchased wood, Theme 19 illustrates a misconceived notion that LPG is more expensive than wood because wood is purchased continuously in small amounts versus the cost of a 25-pound cylinder of LPG, making it difficult to estimate monthly costs. In many parts of the world, people collect free wood fuel; however, in regions of Guatemala where there are few communities or privately held forests, many households purchase wood. In the Jalapa Department, we conducted a rapid assessment in 404 homes in 2016 before starting the HAPIN trial. Among the 93% of household who used wood fuel, households spent an average of U.S. $21 monthly to purchase wood (Campbell, n.d.) Among the 800 households recruited and randomized in 2018–2019 during the HAPIN trial, at baseline 457 (57%) reported buying firewood, with a median cost of US $26. During the HAPIN trial, the cost of a 25-pound tank of LPG gas in Guatemala was $12.80 and the median monthly use of cylinders per household is 2.8 cylinders, or U.S. $36 per month (Mollinedo & McCracken, n.d.).

### Health Outcomes

While the HAPIN trial assesses intervention effects on health benefits, the inherent advantages of using LPG stoves to reduce diseases and discomforts associated with biomass smoke were not described by participants. Firewood, for example, was considered natural, and therefore healthier than LPG. Participants described elders as having diseases and discomforts as part of a normal body “weakening” due to their age and not a consequence of indoor air pollution exposure (Table 3). Themes 13, 14, 16, and 20 focus on how cleaner and efficient cooking technologies and fuels can improve health (Table 2).

### LPG Is “Not Natural”

Even complex ideas, such as misperceptions of where LPG comes from, were illustrated. This was necessary because participants mentioned that LPG was “unnatural” and “smelled poisonous,” unlike wood (Table 3). Participants stated that LPG is a manufactured product that makes food poisonous, as opposed to firewood (Table 2, Theme 14). Explaining that LPG is odorless and therefore the gas company adds mercaptan, which smells bad, is a way to alert families that there is a leak (Table 2, Themes 3 and 4). We developed a message that gas is a fossil fuel that comes from the ground and is processed and delivered into the home in cylinders for cooking use (Table 2, Theme 24).

### Inspirational and Aspirational Testimonial Messages

During our FGDs, participant testimonials were viewed as inspirational by other participants. Therefore, we chose quotes that supported visual messages on the calendar and flip chart. For Indigenous communities, daily cooking practices are customarily embedded in social relations of responsibility, reciprocity, and knowledge transmission. Positive remarks by women who participated in the pilot study were used to reverse the negative sentiments of cultural appropriation in which the new technology (the LPG stove) and new cooking practices acquire significance within the existing sociocultural context (Theme 18).

Time-savings was an important aspirational message, “with the time I save now that I don’t have to collect wood, I make my house pretty, there is no soot, and I have time to feed my animals and water my flowers.” Cleanliness was another aspiration (Table 2, Theme 8) and was reflected in messages like “my brothers and sisters are happy because my mother doesn’t suffer from cooking with wood” and “my children don’t get covered with smoke and soot anymore. My (older) children are now independent and cook (too).”

Testimonial messages, tied together with visual images, is not only a way of gaining insight into how people process visual information but also allows the graphic designer to capture emotional expressions, facial, and body language and spatial relations. This allowed us to modify the draft images based on open-ended commentaries from participants until consistency was reached (Figure 1).

## Discussion

Social phenomenology framed our understanding of people engaging in their everyday world (Desjarlais & Throop, 2011). Testimonials can overcome the sense of “cultural appropriation” from an anthropological perspective (Hage, 2015), especially when adopting new ways of cooking might be seen as a loss of tradition. People who had experience using LPG stoves provided diffusion of ideas, rather than cultural appropriation, which can exert a powerful influence on social networks (Mills & Peeples, 2019). We used qualitative methods, including participant observation, in-depth interviews, and focus group discussions to elicit themes and develop messages to create a calendar with 12 themes and a flip chart with all 24 themes. The 12 topics chosen for the calendar address issues that are crucially important to overcome fear or reluctance, two main aspects that consistently appeared during the pilot study, to provide people with confidence instead. Several of the themes we found are similar to other studies, including fear and reluctance to use LPG in Cameroon (Stanistreet et al., 2019); traditional foods are better when cooked on wood stoves in Peru (Hollada et al., 2017); the inherent contrast between wood versus LPG in Guatemala (Thompson et al., 2018); sustainability and accessibility of fuel in Cameroon (Pope et al., 2018); lack of an association between health outcomes and household air pollution in Kenya and Ethiopia (Loo et al., 2016; Tamire et al., 2018); and the need for inspirational and aspirational messages, instead of negative messaging in Senegal (Hooper et al., 2018). Because the HAPIN study provides free LPG stoves and fuel, and our focus group participants were members of the pilot stove and fuel distribution program, we did not hear common themes described in other studies, such as the need for reliable distribution of LPG equipment and cylinder refills (Ronzi et al., 2019).

Studies have explored visual messages about environmental health themes, but no prior published studies have used calendar messages about household air pollution. Photovoice methods, where participants photograph and then reflect on their lived experiences, were used in Cameroon to document barriers to uptake of LPG cooking (Ronzi et al., 2019). One recent study in Nepal used art during focus groups to illustrate women’s lived experience of exposure to household indoor air pollution (Devakumar et al., 2018). A local artist produced images that depicted perceived health problems as well as solutions, such as improved ventilation. Our current study goes beyond a description of the problem as done in these two studies; our calendar messages are used to promote behavior change, increasing adherence to the LPG stove intervention. We found one example of a seasonal calendar developed in Australia to promote aquatic resource management based on the ecological knowledge of Ngan’gi Aboriginal people (Woodward & Taggart, 2019). While the intent of the seasonal calendar was used differently than ours, both incorporated local knowledge of seasons, climate change, and natural resource management.

These materials are being used in intervention households in the HAPIN trial in Guatemala, where family members are instructed on cleaning the stove, using the LPG cylinder safety valve, lighting and regulating flame strength, recognizing and responding to gas leaks, and reporting malfunctioning stoves and cylinders. At installation, the calendar is reviewed with the family and left in the household. At subsequent visits, themes are reinforced and cylinder refills are marked on the calendar. If our field team detects biomass stove use in intervention homes (e.g., traditional stoves lit or smoldering at home visits, temperature sensors on traditional stoves showing evidence of use; Wilson et al., 2020), a behavioral interventionist visits the households to reinforce exclusive LPG use, using pertinent messages in the flip chart to guide discussion with family members.

LPG fuel cost is the primary barrier for low-income households in countries that do not subsidize LPG fuel, such as Guatemala (Thompson et al., 2018); cost barriers have been well documented in other countries (Gould & Urpelainen, 2018; Pillarisetti et al., 2019; Puzzolo et al., 2016; Quinn et al., 2018). Household income, fuel prices, and subsidies are the primary determinants of sustained use of LPG in poor households (Kojima, 2011). Where national governments have embarked on programs to subsidize clean fuels, like in India (Mani et al., 2020), Ecuador (Gould & Urpelainen, 2018) and Peru (Pollard et al., 2018), LPG stoves have achieved sustained adoption among the poor. Because the HAPIN trial was an efficacy trial, we did not focus on posttrial sustained use of LPG. This is, however, a focus of our planned follow-up study, HAPIN II, which will assess sustained use of the LPG stove, including refill consumption, wood collection (time and cost) as well as changes in household air pollution.

There are several strengths of this study. First, this was a collaboration between researchers, study participants and a graphic designer to discover themes and develop visual messages that represent the daily lives of the study area population, many of whom were Xinca indigenous communities. In this sense, we agree with Pink (2006) who argued “. . . in any project a researcher should attend not only to the internal ‘meanings’ of an image, but also to how the image was produced and how it is made meaningful by its viewers” (p. 186). Second, our formative phase participants had 2 months of experience with exclusive LPG use. As new users, they expressed fears they had before using LPG, and said that positive safety messages would be more effective than messages that provoke fear. Third, we use images as a messaging tool in the HAPIN trial conducted in rural communities of Jalapa to reinforce the sustained use of LPG stoves and fuel. Each image systematizes problem solving around a different topic. As Liebenberg (2009) states, “experiences and meanings become tangible through visual representation and may be understood in ways that other conventional forms of communication may not necessarily allow” (p. 445). Four, the new messages can be more effective when they have been designed within similar audiences and pinpoint the components that are associated with their own concerns. These components merited the design of an in-depth formative phase to ensure adherence by overcoming cultural barriers, such as cooking practices, taking into consideration that economic barriers are not a factor during the HAPIN trial. As the “one size fits all” approach never works, it is more efficient to focus the strategy on people who share the same lifestyles, environmental settings, motivations, perceptions, and social values.

There are several limitations to interpretation of our findings. First, a calendar with appealing visual messages will not be instructional without people or processes that permit participants to replace traditional practices with new practices. To address this, a behavioral interventionist visits intervention households in the HAPIN trial to address persistent traditional stove use. This field worker uses the calendar or flip chart to identify barriers and then reinforce LPG use, for instance, demonstrating how to prepare a specific traditional meal on the LPG stove. Second, while we have 24 themes, 12 of them are not in the calendar and may only be addressed because a solid fuel stove was used in an intervention household. Therefore, some complex themes such as “where does gas come from?” may not be discussed, unless a household member asks that question. Third, given the short time frame of the pilot study (2 months), and the long-term behavioral changes needed to adopt and exclusively use LPG, participant responses may not have provided all the potential and positive aspects of cooking with cleaner fuels. We estimate that within the 400 Guatemalan intervention households participating in the HAPIN trial over 18 months, we will be able to fully characterize the behavioral aspects that determine adoption and sustained use of LPG in the future. Fourth, findings may not be generalizable to other communities, or other type of solid fuel use, like animal dung. Some of the concerns, like a smoky kitchen from solid fuel use, might be relevant. However, the results of the visual messages are likely not applicable to different ethnic groups and geographic areas in Guatemala since clothing, house materials, and stove types were drawn to suit the rural people of these Xinca communities. Finally, during the HAPIN trial, we may uncover additional household concerns and may develop new messages to assuage these concerns, but we did not do that in the present study.

### Implications

Peoples’ experiences are an integral part of the complex whole—daily cooking involves choices, culinary habits, and availability of commodities, overlapping with experiences based on social conditions. This holistic approach helps us understand how changes influence “cooking systems” (Sobreira et al., 2018). External forces, like the introduction of a new stove technology, will necessarily lead to a recontextualization of beliefs and behaviors. It is important to address all underlying reasons, including “subjective feelings” toward LPG stoves and activities related to cooking behaviors. Future directions in the field should include an examination of the context-specific barriers and enablers to adopt and sustain the use of clean cooking (Puzzolo et al., 2016). One of the biggest barriers to use of clean stoves is national policies that support fuel subsidies or promote clean cookstoves, and research is needed to support policy development (Puzzolo et al., 2016; Smith et al., 2014; Stanistreet et al., 2019). This formative research did not focus on policy implications, but the aim of the HAPIN trial is to provide rigorous evidence to policy makers, showing that exclusive use of LPG stoves will show positive health effects in children and other household members (Barr et al., 2020; Clasen et al., 2020; Johnson et al., 2020).

Cooking with LPG and other clean fuels can dramatically reduce PM_2.5_ (particulate matter <2.5 µm in diameter) from household air pollution (Steenland et al., 2018) and may improve health of children and women in developing countries where solid fuels are used for cooking. Yet, adherence to clean cookstoves will be low if participants do not see the value of the new stove. The challenge to confronting deeply rooted beliefs and habits is to understand what motivates people to change behavior when faced with a new technology. We identified information gaps that could prevent new users from exclusively using the LPG stove. Our visual messages prioritized how to reduce harm when using LPG safely and how LPG can improve health, well-being and the environment compared with wood stove use. The images drew on the strengths of LPG and the disadvantages of firewood, addressing fears and doubts that prevent LPG use, as well as exploring the integration of new cooking behaviors.

### Conclusions

Visual messages using pictorial data play an important role in reinforcing messages and make information comprehensible to lower literacy groups. People’s choices and preferences around stoves and fuels are multifaceted and depend not only on “affordability,” “accessibility,” and “availability.” Concepts such as “fear,” “better,” and “healthier” may factor into choices. The HAPIN trial provides free LPG fuel for 18 months, thus addressing “affordability,” “accessibility,” and “availability.” However, new users must be convinced that there are both tangible and intangible benefits when households abandon their traditional solid fuel stoves. The intent of the flip chart and the calendar is to address fears, motivations, and aspirations to support the exclusive use of LPG during the HAPIN trial. Translating peoples’ experiences, ideas, and perceptions into visual images creates a representation of LPG stove use. While these messages are not generalizable to all settings, future work will be done to evaluate the importance of context-specific reinforcing messages during the HAPIN trial.

## Supplemental Material

sj-docx-1-heb-10.1177_1090198121996280 – Supplemental material for Developing Visual Messages to Support Liquefied Petroleum Gas Use in Intervention Homes in the Household Air Pollution Intervention Network (HAPIN) Trial in Rural Guatemala Supplemental material, sj-docx-1-heb-10.1177_1090198121996280 for Developing Visual Messages to Support Liquefied Petroleum Gas Use in Intervention Homes in the Household Air Pollution Intervention Network (HAPIN) Trial in Rural Guatemala by Mayarí Hengstermann, Anaité Díaz-Artiga, Roberto Otzóy-Sucúc, Ana Laura Maria Ruiz-Aguilar and Lisa M. Thompson in Health Education & Behavior

## References

[bibr1-1090198121996280] Bagnoli A. (2009). Beyond the standard interview: The use of graphic elicitation and arts-based methods. Qualitative Research, 9(5), 547–570. 10.1177/1468794109343625

[bibr2-1090198121996280] Barnes B. Mathee A. Thomas E. (2011). The impact of health behaviour change intervention on indoor air pollution indicators in the rural North West Province, South Africa. Journal of Energy in Southern Africa, 22(3), 35–44. 10.17159/2413-3051/2011/v22i3a3220

[bibr3-1090198121996280] Barr D. B. Puttaswamy N. Jaacks L. M. Steenland K. Rajkumar S. Gupton S. Ryan P. B. Balakrishnan K. Peel J. L. Checkley W. Clasen T. Clark M. L. (HAPIN Investigative Team). (2020). Design and rationale of the biomarker center of the Household Air Pollution Intervention Network (HAPIN) trial. Environmental Health Perspectives, 128(4), 047010. 10.1289/EHP5751 PMC7228115 32347765

[bibr4-1090198121996280] Campbell D. (n.d.). Rapid assessment of fuel use and household air pollution in rural Jalapa, Guatemala [Unpublished raw data]. Universidad del Valle de Guatemala.

[bibr5-1090198121996280] Chen P. (1989). Visual communication materials for rural audiences: Re-orienting artists and copy-writers. Development Communication Report, 66, 4–5. 12283152

[bibr6-1090198121996280] Clasen T. Checkley W. Peel J. Balakrishnan K. McCracken J. P. Rosa G. Thompson L. M. Boyd Barr D. Clark M. Johnson M. Waller L. A. Jaacks L. M. Steeland K. Miranda J. J. Chang H. Kim D. Y. Rosenthal J. , & HAPIN Investigators. (2020). Design and rationale of the Household Air Pollution Intervention Network (HAPIN) study: A multi-country randomized controlled trial to assess the effect of liquefied petroleum gas stove and continuous fuel distribution on household air pollution and health. Environmental Health Perspectives, 128(4), 47008. 10.1289/EHP6407 32347766 PMC7228119

[bibr7-1090198121996280] Copeland A. J. Agosto D. E. (2012). Diagrams and relational maps: The use of graphic elicitation techniques with interviewing for data collection, analysis, and display. International Journal of Qualitative Methods, 11(5), 513–533. 10.1177/160940691201100501

[bibr8-1090198121996280] Desjarlais R. Throop J. (2011). Phenomenological approaches in anthropology. Annual Review of Anthropology, 40(1), 87–102. 10.1146/annurev-anthro-092010-153345

[bibr9-1090198121996280] Devakumar D. Qureshi Z. Mannell J. Baruwal M. Sharma N. Rehfuess E. Saville N. M. Manandhar D. S. Osrin D. (2018). Women’s ideas about the health effects of household air pollution, developed through focus group discussions and artwork in southern Nepal. International Journal of Environmental Research and Public Health, 15(2), 248. 10.3390/ijerph15020248 29389909 PMC5858317

[bibr10-1090198121996280] Evans W. D. Johnson M. Jagoe K. Charron D. Young B. N. Rahman A. S. M. M. Omolloh D. Ipe J. (2018). Evaluation of behavior change communication campaigns to promote modern cookstove purchase and use in lower middle income countries. International Journal of Environmental Research and Public Health, 15(1), 11. 10.3390/ijerph15010011 PMC5800111 29271949

[bibr11-1090198121996280] Fandiño-Del-Rio M. Goodman D. Kephart J. L. Williams K. N. Moazzami M. Fung E. C. Koehler K. Davila-Roman V. G. Lee K. A. Nangia S. Harvey S. A. Steenland K. Gonzales G. F. Checkley W. , & CHAP Trial Investigators. (2017). Effects of a liquefied petroleum gas stove intervention on pollutant exposure and adult cardiopulmonary outcomes (CHAP): Study protocol for a randomized controlled trial. Trials, 18(1), 518. 10.1186/s13063-017-2179-x 29100550 PMC5670728

[bibr12-1090198121996280] Fedak K. M. Good N. Walker E. Clark M. L. L’Orange C. Volckens J. Peel J. L. (2019). An expert survey on the material types used to start cookstoves. Energy for Sustainable Development: The Journal of the International Energy Initiative, 48, 59–66. 10.1016/j.esd.2018.1.001 31598056 PMC6784844

[bibr13-1090198121996280] Goodwin N. J. O’Farrell S. E. Jagoe K. Rouse J. Roma E. Biran A. Finkelstein E. A. (2015). Use of behavior change techniques in clean cooking interventions: A review of the evidence and scorecard of effectiveness. Journal of Health Communication, 20(1 suppl.), 43–54. 10.1080/10810730.2014.1002958 25839202

[bibr14-1090198121996280] Gould C. F. Urpelainen J. (2018). LPG as a clean cooking fuel: Adoption, use, and impact in rural India. Energy Policy, 122, 395–408. 10.1016/j.enpol.2018.07.042 PMC7314235 32581420

[bibr15-1090198121996280] Guillemin M. (2004). Understanding illness: Using drawings as a research method. Qualitative Health Research, 14(2), 272–289. 10.1177/1049732303260445 14768462

[bibr16-1090198121996280] Hage G. (2015). Alter-politics. Critical anthropology and the radical imagination. Social Anthropology, 25(1), 115–117. 10.1111/1469-8676.12382

[bibr17-1090198121996280] Harper D. (2002). Talking about pictures: A case for photo elicitation. Visual Studies, 17(1), 13–26. 10.1080/14725860220137345

[bibr18-1090198121996280] Health Effects Institute. (2019). State of global air 2019: A special report on global exposure to air pollution and its disease burden. https://www.stateofglobalair.org/sites/default/files/soga_2019_report.pdf

[bibr19-1090198121996280] Hollada J. Williams K. N. Miele C. H. Danz D. Harvey S. A. Checkley W. (2017). Perceptions of improved biomass and liquefied petroleum gas stoves in Puno, Peru: Implications for promoting sustained and exclusive adoption of clean cooking technologies. International Journal of Environmental Research and Public Health, 14(2), 182. 10.3390/ijerph14020182 28208813 PMC5334736

[bibr20-1090198121996280] Hooper L. G. Dieye Y. Ndiaye A. Diallo A. Sack C. S. Fan V. S. Neuzil K. M. Ortiz J. R. (2018). Traditional cooking practices and preferences for stove features among women in rural Senegal: Informing improved cookstove design and interventions. PLoS ONE, 13(11), e0206822. 10.1371/journal.pone.0206822 PMC6245512 30458001

[bibr21-1090198121996280] Houts P. S. Doak C. C. Doak L. G. Loscalzo M. J. (2006). The role of pictures in improving health communication: A review of research on attention, comprehension, recall, and adherence. Patient Education Counseling, 61(2), 173–190. 10.1016/j.pec.2005.05.004 16122896

[bibr22-1090198121996280] Jensen J. D. King A. J. Carcioppolo N. Davis L. (2012). Why are tailored messages more effective? A multiple mediation analysis of breast cancer screening intervention. Journal of Communication, 62(5), 851–868. 10.1111/j.1460-2466.2012.01668.x 26405350 PMC4578294

[bibr23-1090198121996280] Jin Y. Ma X. Chen X. Cheng Y. Baris E. Ezzati M. (2006). Exposure to indoor air pollution from household energy use in rural China: The interactions of technology, behavior, and knowledge in health risk management. Social Science & Medicine, 62(12), 3161–3176. 10.1016/j.socscimed.2005.11.029 16426715

[bibr24-1090198121996280] Johnson M. A. Steenland K. Piedrahita R. Clark M. L. Pillarisetti A. Balakrishnan K. Peel J. L. Naeher L. P. Liao J. Wilson D. Sarnat J. Underhill L. J. Burrowes V. McCracken J. P. Rosa G. Rosenthal J. Sambandam S. de Leon O. Kirby M. A. Kearns K. Checkley W. Clasen T. HAPIN Investigators. (2020). Air pollutant exposure and stove use assessment methods for the Household Air Pollution Intervention Network (HAPIN) trial. Environmental Health Perspectives, 128(4), 047009. 10.1289/EHP6422 PMC7228125 32347764

[bibr25-1090198121996280] Kojima M. (2011). The role of liquefied petroleum gas in reducing energy poverty (No. 25; Extractive Industries for Development Series). World Bank.

[bibr26-1090198121996280] Kreuter M. W. Strecher V. J. Glassman B. (1999). One size does not fit all: The case for tailoring print materials. Annals of Behavioral Medicine, 21(4), 276–283. 10.1007/BF02895958 10721433

[bibr27-1090198121996280] Liebenberg L. (2009). The visual image as discussion point: Increasing validity in boundary crossing research. Qualitative Research, 9(4), 441–467. 10.1177/1468794109337877

[bibr28-1090198121996280] Lipkus I. M. (2007). Numeric, verbal, and visual formats of conveying health risks: Suggested best practices and future recommendations. Medical Decision Making, 27(5), 696–713. 10.1177/0272989X07307271 17873259

[bibr29-1090198121996280] Lipkus I. M. Hollands J. G. (1999). The visual communication of risk. Journal of the National Cancer Institute Monographs, 25, 149–163. 10.1093/oxfordjournals.jncimonographs.a024191 10854471

[bibr30-1090198121996280] Loo J. D. Hyseni L. Ouda R. Koske S. Nyagol R. Sadumah I. Bashin M. Sage M. Bruce N. Pilishvili T. Stanistreet D. (2016). User perspectives of characteristics of improved cookstoves from a field evaluation in western Kenya. International Journal of Environmental Research and Public Health, 13(2), 167. 10.3390/ijerph13020167 26828505 PMC4772187

[bibr31-1090198121996280] Mani S. Jain A. Tripathi S. Gould C. F. (2020). The drivers of sustained use of liquified petroleum gas in India. Nature Energy, 5(6), 450–457. 10.1038/s41560-020-0596-7 PMC7384753 32719732

[bibr32-1090198121996280] Mills B. J. Peeples M. A. (2019). Reframing diffusion through social network theory. In Harry K. G. Roth B. J. (Eds.), Interaction and connectivity in the greater Southwest (pp. 40–62). University Press of Colorado.

[bibr33-1090198121996280] Mollinedo E. McCracken J. P. (n.d.). Baseline sociodemographic and household fuel use in rural Jalapa: The HAPIN trial [Unpublished Raw Data]. Universidad del Valle de Guatemala.

[bibr34-1090198121996280] Oluwole O. Ana G. R. Arinola G. O. Wiskel T. Falusi A. G. Huo D. Olopade C. O. (2013). Effect of stove intervention on household air pollution and the respiratory health of women and children in rural Nigeria. Air Quality, Atmosphere & Health, 6(3), 553–561. 10.1007/s11869-013-0196-9

[bibr35-1090198121996280] Pillarisetti A. Ghorpade M. Madhav S. Dhongade A. Roy S. Balakrishnan K. Sankar S. Patil R. Levine D. I. Juvekar S. Smith K. R. (2019). Promoting LPG usage during pregnancy: A pilot study in rural Maharashtra, India. Environment International, 127, 540–549. 10.1016/j.envint.2019.04.017 30981912 PMC7213905

[bibr36-1090198121996280] Pink S. (2006). The future of visual anthropology: Engaging the senses. Routledge.

[bibr37-1090198121996280] Pollard S. L. Williams K. N. O’Brien C. J. Winiker A. Puzzolo E. Kephart J. L. Fandiño-Del-Rio M. Tarazona-Meza C. Grigsby M. Chiang M. Checkley W. (2018). An evaluation of the Fondo de Inclusión Social Energético program to promote access to liquefied petroleum gas in Peru. Energy for Sustainable Development: The Journal of the International Energy Initiative, 46, 82–93. 10.1016/j.esd.2018.06.001 30364502 PMC6197055

[bibr38-1090198121996280] Pope D. Bruce N. Higgerson J. Hyseni L. Stanistreet D. MBatchou B. Puzzolo E. (2018). Household determinants of liquified petroleum gas (LPG) as a cooking fuel in SW Cameroon. EcoHealth, 15, 729–743. 10.1007/s10393-018-1367-9 30276494 PMC6267519

[bibr39-1090198121996280] Prochaska J. O. Norcross J. C. (2001). Stages of change. Psychotherapy: Theory, Research, Practice, Training, 38(4), 443–448. 10.1037/0033-3204.38.4.443

[bibr40-1090198121996280] Puzzolo E. Pope D. Stanistreet D. Rehfuess E. A. Bruce N. G. (2016). Clean fuels for resource-poor settings: A systematic review of barriers and enablers to adoption and sustained use. Environmental Research, 146, 218–234. 10.1016/j.envres.2016.01.002 26775003

[bibr41-1090198121996280] Quinn A. Bruce N. Puzzolo E. Dickinson K. Sturke R. Jack D. W. Mehta S. Shankar A. Sherr K. Rosenthal J. (2018). An analysis of efforts to scale up clean household energy for cooking around the world. Energy for Sustainable Development: The Journal of the International Energy Initiative, 46, 1–10. 10.1016/j.esd.2018.06.011 30886466 PMC6419773

[bibr42-1090198121996280] Ronzi S. Puzzolo E. Hyseni L. Higgerson J. Stanistreet D. Hugo M. N. B. Bruce N. G. Pope D. (2019). Using photovoice methods as a community-based participatory research tool to advance uptake of clean cooking and improve health: The LPG adoption in Cameroon evaluation studies. Social Science & Medicine, 228, 30–40. 10.1016/j.socscimed.2019.02.044 PMC7081729 30875542

[bibr43-1090198121996280] Ross N. J. Renold E. Holland S. Hillman A. (2009). Moving stories: Using mobile methods to explore the everyday lives of young people in public care. Qualitative Research, 9(5), 605–623. 10.1177/1468794109343629

[bibr44-1090198121996280] Ruiz-Mercado I. Canuz E. Walker J. L. Smith K. R. (2013). Quantitative metrics of stove adoption using stove use monitors (SUMs). Biomass & Bioenergy, 57, 136–148. 10.1016/j.biombioe.2013.07.002 PMC4170739 25258474

[bibr45-1090198121996280] Schutz A. (1967). The phenomenology of the social world. Northwestern University Press.

[bibr46-1090198121996280] Sharma M. Dasappa S. (2017). Emission reduction potentials of improved cookstoves and their issues in adoption: An Indian outlook. Journal of Environmental Management, 204(Part 1), 442–453. 10.1016/j.jenvman.2017.09.018 28917179

[bibr47-1090198121996280] Shupler M. Hystad P. Birch A. Miller-Lionberg D. Jeronimo M. Arku R. E. Chu Y. L. Mushtaha M. Heenan L. Rangarajan S. Seron P. Lanas F. Cazor F. Lopez-Jaramillo P. Camacho P. A. Perez M. Yeates K. West N. Ncube T. , . . . on behalf of the PURE-AIR study. (2020). Household and personal air pollution exposure measurements from 120 communities in eight countries: Results from the PURE-AIR study. The Lancet Planetary Health, 4(10), e451–e462. 10.1016/S2542-5196(20)30197-2 PMC7591267 33038319

[bibr48-1090198121996280] Smith K. R. Bruce N. Balakrishnan K. Adair-Rohani H. Balmes J. Chafe Z. Dherani M. Hosgood H. D. Mehta S. Pope D. Rehfuess E. HAP CRA Risk Expert Group. (2014). Millions dead: How do we know and what does it mean? Methods used in the comparative risk assessment of household air pollution. Annual Review of Public Health, 35(1), 185–206. 10.1146/annurev-publhealth-032013-182356 24641558

[bibr49-1090198121996280] Sobreira L. B. Garavello M. E. P. E. Nardoto G. B. (2018). Anthropology of food: An essay on food transition and transformations in Brazil. Journal of Food, Nutrition Population Health, 2(1), 9. 10.21767/2577-0586.10039

[bibr50-1090198121996280] Stanistreet D. Hyseni L. Puzzolo E. Higgerson J. Ronzi S. Anderson de Cuevas R. Adekoje O. Bruce N. Mbatchou Ngahane B. Pope D. (2019). Barriers and facilitators to the adoption and sustained use of cleaner fuels in southwest Cameroon: Situating ‘lay’ knowledge within evidence-based policy and practice. International Journal of Environmental Research and Public Health, 16(23), 4702. 10.3390/ijerph16234702 PMC6926764 31779156

[bibr51-1090198121996280] Steenland K. Pillarisetti A. Kirby M. Peel J. Clark M. Checkley W. Chang H. H. Clasen T. (2018). Modeling the potential health benefits of lower household air pollution after a hypothetical liquified petroleum gas (LPG) cookstove intervention. Environment International, 111, 71–79. 10.1016/j.envint.2017.11.018 29182949 PMC5801118

[bibr52-1090198121996280] Tamire M. Addissie A. Skovbjerg S. Andersson R. Lärstad M. (2018). Socio-cultural reasons and community perceptions regarding indoor cooking using biomass fuel and traditional stoves in rural Ethiopia: A qualitative study. International Journal of Environmental Research and Public Health, 15(9), 2035. 10.3390/ijerph15092035 PMC6164706 30231480

[bibr53-1090198121996280] Thompson L. M. Hengstermann M. Weinstein J. R. Diaz-Artiga A. (2018). Adoption of liquefied petroleum gas stoves in Guatemala: A mixed-methods study. EcoHealth, 15(4), 745–756. 10.1007/s10393-018-1368-8 30229372 PMC6265077

[bibr54-1090198121996280] Thurber M. C. Warner C. Platt L. Slaski A. Gupta R. Miller G. (2013). To promote adoption of household health technologies, think beyond health. American Journal of Public Health, 103(10), 1736–1740. 10.2105/AJPH.2013.301367 23948003 PMC3780735

[bibr55-1090198121996280] Tun K. M. Win H. Ohnmar T. Zaw A. K. M. T. Myat K. K. S. Kyi S. Lwin T. T. (2005). Indoor air pollution: Impact of intervention on acute respiratory infection (ARI) in under-five children. Regional Health Forum, 9(1), 30–36.

[bibr56-1090198121996280] Williams K. Thompson L. M. Sakas Z. Hengstermann M. Quinn A. Diaz-Artiga A. Thangavel G. Puzzolo E. Rosa G. Balakrishnan K. Peel J. Checkley W. Clasen T. Miranda J. Rosenthal J. Harvey S. , & HAPIN investigators. (2020). Designing a comprehensive behavior change framework to promote and monitor exclusive use of liquefied petroleum gas stoves for the Household Air Pollution Intervention Network (HAPIN) trial. BMJ Open, 10(9). 10.1136/bmjopen-2020-037761 PMC7526279 32994243

[bibr57-1090198121996280] Wilson D. L. Williams K. N. Pillarisetti A. (2020). An integrated sensor data logging, survey, and analytics platform for field research and its application in HAPIN, a multi-center household energy intervention. Sustainability, 12(5), 1805. 10.3390/su12051805

[bibr58-1090198121996280] Woodward E. Taggart P. M. M. (2019). Co-developing Indigenous seasonal calendars to support “healthy Country, healthy people” outcomes. Global Health Promotion, 26(3 Suppl.), 26–34. 10.1177/1757975919832241 30964407

[bibr59-1090198121996280] World Health Organization. (2014). WHO indoor air quality guidelines: Household fuel combustion. https://www.who.int/airpollution/guidelines/household-fuel-combustion/IAQ_HHFC_guidelines.pdf 25577935

[bibr60-1090198121996280] Zhou Z. Jin Y. Liu F. Cheng Y. Kang J. Ezzati M. (2006). Community effectiveness of stove and health education interventions for reducing exposure to indoor air pollution from solid fuels in four Chinese provinces. Environmental Research Letters, 1(1), 1014010. 10.1088/1748-9326/1/1/014010

