# Supplementary material for: Developing Visual Messages to Support Liquefied Petroleum Gas Use in Intervention Homes in the Household Air Pollution Intervention Network (HAPIN) Trial in Rural Guatemala
Source: Health Educ Behav. 2021 Mar 18;48(5):651–69. doi: 10.1177/1090198121996280 (PMC10666197; doi:10.1177/1090198121996280)
Supplement: sj-docx-1-heb-10.1177_1090198121996280 – Supplemental material for Developing Visual Messages to Support Liquefied Petroleum Gas Use in Intervention Homes in the Household Air Pollution Intervention Network (HAPIN) Trial in Rural Guatemala [file sj-docx-1-heb-10.1177_1090198121996280.docx]

Based on previous research and literature review, the research team pre-identified a few themes that concentrated on all the possible setbacks to explore the constraints of using LPG, such as the infrastructure, affordability, and the experiences and perspectives of the participants.

Cooking behaviors are multi-dimensional and even linked to unexpected dynamics and issues. For this reason, the HAPIN-trial team made use of the HAPIN BEC in-depth interview guide during the scoping phase, exploring the variety of aspects that potentially related to cooking behaviours, sociocultural and environmental contexts among the target population. This open-ended guide addressed most of the issues that were identified beforehand but it was flexible when collecting the qualitative data during the observations and interviews, allowing the engagement of conversations with the people that were being interviewed, generating a narrative that permitted the expansion of aspects that might have been new for the researcher as well as relevant for the participants. Thus, the interview guide was on its own limited, yet allowed the involvement of a significant array of aspects that the participants elaborated on or explained in-depth.

The phenomenological and empirical approach deliberately created the potential to include a rich narrative and description of the diverse cooking behaviours as an ongoing process. The method was broken down into three parts: data collection, data reduction, validation. All the data was transcribed and converted into a word document and later translated into an excel table. The process firstly achieved this by transcribing, sorting and then coding, creating a thematic that provided explanations to each context or issue, following the procedure (1) transcription of the data into word, (2) reducing, sorting and coding data by taking the interview’s responses using HyperRESEARCH Software, and (3) translating quotations that responded to a single topic, subject or experience, using an excel table. Each interview and fieldwork notes were a single file, then grouped by communities, later on by main and sub-codes to finally generate networks. The pre-identified themes -along with the new ones- created the 24 ‘visual messages’

For example, when asking ‘*what type of stoves do people use or have used before, besides the traditional stove’*, one of the responses was:

“…*I only use a firewood stove. We have an electric stove of two burners that we got when we married but I only use it to cook eggs, an instant soup or boil water for coffee. You can’t use it for ‘proper’ cooking, like preparing beans or tortillas. For that you need a big stove and a direct flame, that’s only possible if you use firewood. Besides, the pots we use for that are really big, so you can’t put these on the burners. The pots are made of clay, you know? And that’s what we use to prepare beans. For those gas or electric stoves, you need tin pots and pans and those are not only expensive but also those are not good to cook beans or prepare atole. Even if you have a gas or electric stove you need a firewood stove. Now my electric stove isn’t working, just one burner but if it gets broken, I think we won’t get a new one. My husband doesn’t like it although I do because in the morning it’s really easy to boil water and reheat the food; you don’t need to wake up too early to prepare the firewood to do that. It saves you time…I really like it. It has a nice color. However, I wouldn’t use a gas stove. I’m too afraid. You always hear things like houses that have been burned down due to explosions or children that suffered from severe burns. I think a gas stove is just not safe and way too expensive…”*

In this transcript’s excerpt, we could identify more ‘topics’ than the one being asked. The main topic was “experiences with other modern stoves”. Guided by the phenomenological approach, participants elaborated in detail their responses even if the data seemed at the time not to be ‘relevant’ for the researcher, but which increased the understanding of practices and perceptions.

The process of coding works as followed:

Code: Experiences with other stoves (main question)

“…*I only use a firewood stove. We have an electric stove of two burners that we got when we married but I only use it to cook eggs, an instant soup or boil water for coffee. You can’t use it for ‘proper’ cooking, like preparing beans or tortillas. For that you need a big stove and a direct flame; that’s only possible if you use firewood. Besides, the pots we use for that are really big, so you can’t put these on the burners. The pots are made of clay, you know? And that’s what we use to prepare beans. For those gas or electric stoves, you need tin pots and pans and those are not only expensive but also those are not good to cook beans or prepare atole. Even if you have a gas or electric stove you need a firewood stove. Now my electric stove isn’t working, just one burner but if it gets broken, I think we won’t get a new one. My husband doesn’t like it although I do because in the morning it’s really easy to boil water and reheat the food; you don’t need to wake up too early to prepare the firewood to do that. It saves you time…I really like it. It has a nice color. However, I wouldn’t use a gas stove. I’m too afraid. You always hear things like houses that have been burned down due to explosions or children that suffered from severe burns. I think a gas stove is just not safe and way too expensive…”*

These responses were selected as “types of stoves used”. Then we created other codes that were not initially part of the question, resulting in many and varied codes. Many responses corresponded to more than one code (for example):

Code: Reasons to use other stoves (sub-theme)

“…*I only use a firewood stove. We have an electric stove of two burners that we got when we married but I only use it to cook eggs, an instant soup or boil water for coffee. You can’t use it for ‘proper’ cooking, like preparing beans or tortillas. For that you need a big stove and a direct flame, that’s only possible if you use firewood. Besides the pots we use for that are really big, so you can’t put these on the burners. The pots are made of clay, you know? And that’s what we use to prepare beans. For those gas or electric stoves, you need tin pots and pans and those are not only expensive but also those are not good to cook beans or prepare atole. Even if you have a gas or electric stove you need a firewood stove. Now my electric stove isn’t working, just one burner but if it gets broken, I think we won’t get a new one. My husband doesn’t like it although I do because in the morning it’s really easy to boil water and reheat the food; you don’t need to wake up too early to prepare the firewood to do that. It saves you time… I really like it. It has a nice color. However, I wouldn’t use a gas stove. I’m too afraid. You always hear things like houses that have been burned down due to explosions or children that suffered from severe burns. I think a gas stove is just not safe and way too expensive…”*

Code: Reasons to NOT use other stoves (sub-theme)

“…*I only use a firewood stove. We have an electric stove of two burners that we got when we married but I only use it to cook eggs, an instant soup or boil water for coffee. You can’t use it for ‘proper’ cooking, like preparing beans or tortillas. For that you need a big stove and a direct flame, that’s only possible if you use firewood. Besides the pots we use for that are really big, so you can’t put these on the burners. The pots are made of clay, you know? And that’s what we use to prepare beans. For those gas or electric stoves, you need tin pots and pans and those are not only expensive but also those are not good to cook beans or prepare atole. Even if you have a gas or electric stove you need a firewood stove. Now my electric stove isn’t working, just one burner but if it gets broken, I think we won’t get a new one. My husband doesn’t like it although I do because in the morning it’s really easy to boil water and reheat the food; you don’t need to wake up too early to prepare the firewood to do that. It saves you time… I really like it. It has a nice color. However, I wouldn’t use a gas stove. I’m too afraid. You always hear things like houses that have been burned down due to explosions or children that suffered from severe burns. I think a gas stove is just not safe and way too expensive…”*

Code: What types of food are prepared in non-traditional stoves (main question). Overlapping with the Code: Reasons to use other stoves (sub-theme)

“…*I only use a firewood stove. We have an electric stove of two burners that we got when we married but I only use it to cook eggs, an instant soup or boil water for coffee. You can’t use it for ‘proper’ cooking, like preparing beans or tortillas. For that you need a big stove and a direct flame, that’s only possible if you use firewood. Besides the pots we use for that are really big, so you can’t put these on the burners. The pots are made of clay, you know? And that’s what we use to prepare beans. For those gas or electric stoves, you need tin pots and pans and those are not only expensive but also those are not good to cook beans or prepare atole. Even if you have a gas or electric stove you need a firewood stove. Now my electric stove isn’t working, just one burner but if it gets broken, I think we won’t get a new one. My husband doesn’t like it although I do because in the morning it’s really easy to boil water and reheat the food; you don’t need to wake up too early to prepare the firewood to do that. It saves you time… I really like it. It has a nice color. However, I wouldn’t use a gas stove. I’m too afraid. You always hear things like houses that have been burned down due to explosions or children that suffered from severe burns. I think a gas stove is just not safe and way too expensive…”*

If a code didn’t appear in more than one interview, it was considered irrelevant. For example:

Code: Features of a modern Stove (important) (sub-theme)

“…*I only use a firewood stove. We have an electric stove of two burners that we got when we married but I only use it to cook eggs, an instant soup or boil water for coffee. You can’t use it for ‘proper’ cooking, like preparing beans or tortillas. For that you need a big stove and a direct flame, that’s only possible if you use firewood. Besides the pots we use for that are really big, so you can’t put these on the burners. The pots are made of clay, you know? And that’s what we use to prepare beans. For those gas or electric stoves, you need tin pots and pans and those are not only expensive but also those are not good to cook beans or prepare atole. Even if you have a gas or electric stove you need a firewood stove. Now my electric stove isn’t working, just one burner but if it gets broken, I think we won’t get a new one. My husband doesn’t like it although I do because in the morning it’s really easy to boil water and reheat the food; you don’t need to wake up too early to prepare the firewood to do that. It saves you time… I really like it. It has a nice color. However, I wouldn’t use a gas stove. I’m too afraid. You always hear things like houses that have been burned down due to explosions or children that suffered from severe burns. I think a gas stove is just not safe and way too expensive…”*

Code: Color (non-relevant) (sub-theme)

“…*I only use a firewood stove. We have an electric stove of two burners that we got when we married but I only used it to cook eggs, an instant soup or boil water for coffee. You can’t use it for ‘proper’ cooking, like preparing beans or tortillas. For that you need a big stove and a direct flame, that’s only possible if you use firewood. Besides the pots we use for that are really big, so you can’t put these on the burners. The pots are made of clay, you know? And that’s what we use to prepare beans. For those gas or electric stoves, you need tin pots and pans and those are not only expensive but also those are not good to cook beans or prepare atole. Even if you have a gas or electric stove you need a firewood stove. Now my electric stove isn’t working, just one burner but if it gets broken, I think we won’t get a new one. My husband doesn’t like it although I do because in the morning it’s really easy to boil water and reheat the food; you don’t need to wake up too early to prepare the firewood to do that. It saves you time… I really like it. It has a nice color. However, I wouldn’t use a gas stove. I’m too afraid. You always hear things like houses that have been burned down due to explosions or children that suffered from severe burns. I think a gas stove is just not safe and way too expensive…”*

Code: Perceptions of gas stoves (main question)

“…*I only use a firewood stove. We have an electric stove of two burners that we got when we married but I only use it to cook eggs, an instant soup or boil water for coffee. You can’t use it for ‘proper’ cooking, like preparing beans or tortillas. For that you need a big stove and a direct flame, that’s only possible if you use firewood. Besides the pots we use for that are really big, so you can’t put these on the burners. The pots are made of clay, you know? And that’s what we use to prepare beans. For those gas or electric stoves, you need tin pots and pans and those are not only expensive but also those are not good to cook beans or prepare atole. Even if you have a gas or electric stove you need a firewood stove. Now my electric stove isn’t working, just one burner but if it gets broken, I think we won’t get a new one. My husband doesn’t like it although I do because in the morning it’s really easy to boil water and reheat the food; you don’t need to wake up too early to prepare the firewood to do that. It saves you time… I really like it. It has a nice color. However, I wouldn’t use a gas stove. I’m too afraid. You always hear things like houses that have been burned down due to explosions or children that suffered from severe burns. I think a gas stove is just not safe and way too expensive…”*

We created an open-coding to ensure that every aspect relevant for the participants had been mentioned and not predetermined by the researcher. After this process, we started reducing the codes, by seeing which responses i.e. codes, overlapped and then removing those which appeared only a few times. Codes that had at least 50% percent of responses were considered as ‘thematic’, resulting in 50 codes. Sub-themes were those related topics that were not identified as such when elaborating the interview guide, yet it became a topic when it became specific and was supported by many quotes. Each code is supported by key phrases or words from the interviews. After the data was clustered, we developed networks from main codes to identify which concepts were closer or farther away to other themes. This process is also called “thematic node hierarchy” when using other qualitative research software such as Nvivo. This detailed process eliminates the possibility to oversee aspects of importance, since the quotes contained within the themes can be used as many times as possible. However, the naming of the coding can vary.

This methodology allows organize and structure the data collected phenomenologically, as participants opinions and responses are recorded and coded in detail without any underlying assumptions. The setback of this, is that such data collection and transcription is time consuming, yet important to support all the questions and aspects inquired. It is also possible that during the process of coding, other researchers name the codes differently, according to their own perceptions and opinions. Therefore, consensus among the research team is vitally important when using this methodology.
